# GNIP1 functions both as a scaffold protein and an E3 ubiquitin ligase to regulate autophagy in lung cancer

**DOI:** 10.1186/s12964-022-00936-x

**Published:** 2022-08-30

**Authors:** Feifei Zhou, Yufeng Liu, Wenqian Ai, Yanan Wang, Mingxi Gan, Qingkun Jiang, Tianyu Han, Jian-Bin Wang

**Affiliations:** 1 School of Basic Medical Sciences, Nanchang University, Nanchang, 330031 People’s Republic of China; 2 Jiangxi Institute of Respiratory Disease, The First Affiliated Hospital of Nanchang University, Nanchang, 330006 People’s Republic of China; 3 Department of Oral and Maxillofacial Surgery, First Affiliated Hospital of Nanchang University, Nanchang, 330006 People’s Republic of China

**Keywords:** GNIP1, Autophagy, Non-small cell lung cancer, Proliferation, Migration

## Abstract

**Background:**

Glycogen-Interacting Protein 1 (GNIP1), an E3 ligase, is a member of the tripartite motif (TRIM) family proteins. Current studies on GNIP1 mainly focus on glycogen metabolism. However, the function and molecular mechanisms of GNIP1 in regulating autophagy still remains unclear. This study aimed to investigate the regulatory mechanism of GNIP1 in regulating autophagy in non-small cell lung cancer (NSCLC).

**Methods:**

Crystal violet staining assays were used to evaluate the ability of cell growth and proliferation. Transwell and scratch wound healing assays were used to evaluate the cell migration ability. The protein expressions were measured by western blot and immunohistochemistry. Co-immunoprecipitation assays determined the protein–protein interactions. The in vivo effect of GNIP1 on tumor growth was determined by xenograft assay.

**Results:**

We found that GNIP1 was overexpressed in tumor tissues and the expression level of GNIP1 was related to the poor prognosis and the survival time of NSCLC patients. In non-small cell lung cancer (NSCLC), GNIP1 increased proliferation and migration of cancer cells by promoting autophagy. Mechanistic studies indicated that GNIP1, as a scaffold protein, recruited BECN1 and LC3B to promote the formation of autophagosomes. Besides, GNIP1 mediated the degradation of 14-3-3ζ, the negative regulator of VPS34 complex, thus promoting autophagy. Overexpressing GNIP1 promoted tumorigenesis and enhanced autophagy in xenograft models.

**Conclusion:**

GNIP1 promotes proliferation and migration of NSCLC cells through mediating autophagy, which provides theoretical basis for targeting GNIP1 as anti-cancer drugs.

**Video Abstract**

**Supplementary Information:**

The online version contains supplementary material available at 10.1186/s12964-022-00936-x.

## Background

Autophagy is a conserved biological process associated with many cell functions [1, 2]. Autophagy removes excess organelles and substances and provides a homeostasis for normal cell survival [3, 4]. Dysregulation of autophagy homeostasis is responsible for various human diseases, including cancer [5, 6]. Autophagy can be enhanced by several cancer-related stimuli, for example, oncogenic Ras, cytokines such as TNF, endostatin and radiation [7]. During cancer progression, autophagy not only serves as a tumor promoter to provide energy and substrate for tumor growth, but also serves as a tumor suppressor to clear harmful substances and inhibit tumor growth [8]. Thus, clarifying the exact mechanism of autophagy is of great significance for understanding the crucial role of autophagy in cancer progression and providing guidance for tumor therapy.

Autophagy is a complex process involving three major steps: formation of the autophagosomes; fusion of lysosomes with autophagosomes; degradation of engulfed material by lysosomes. More than 30 autophagy related genes (ATGs) have been found to be involved in this process. Briefly, BECN1 is crucial in the initial autophagosome formation. It forms a complex with hVps34 (Human vacuolar protein sorting 34), also known as PIK3C3 (Phosphatidylinositol-3-kinase class III), to regulate localization of other autophagic proteins [7]. LC3B is involved in the process of autophagosome membrane extension [9, 10]. Sequestosome 1 (SQSTM1) /P62 is a substrate for autophagy, which can interact with LC3 and finally enter lysosome for degradation after being sorted by autophagosomes. In addition, there are also some negative regulatory proteins involved in autophagy. 14-3-3ζ is a phosphoserine/threonine binding protein that regulates autophagy, apoptosis and cell cycle progression, etc. Studies have shown that hVps34 binds to 14-3-3ζ in a PKC-dependent manner, 14-3-3ζ/hVps34 association blocks autophagy by inhibiting Vps34 kinase activity [7, 11].

GNIP1 (Glycogenin Interacting Protein 1), commonly known as TRIM7, is a member of the GNIP1 and TRIM protein family. There are four subtypes of *TRIM7* gene: GNIP1, GNIP2, GNIP3 and TRIM7. Among them, GNIP1 is the longest subtype while TRIM7 is the shortest [12]. GNIP1 was first discovered because it interacted with glycogenin and enhanced its own glycosylation activity [13]. Compared to TRIM7, GNIP1 contains not only RING domain, B-box domain and Coiled-coil domain but also B30.2 domain, which is involved in the regulation of glycogen metabolism [14–16]. So far, there are few studies on GNIP1. Reports have shown that GNIP1 promoted the malignant progression of lung adenocarcinoma by stabilizing RACO-1 [17]. GNIP1, as an E3 ubiquitin ligase, was a novel player in regulating glycogen metabolism in skeletal muscle [18]. And in hepatocellular carcinoma, TRIM7 (GNIP1) promoted malignant proliferation of cancer cells [19]. Our previous studies confirmed that TRIM7 affected lung cancer progression by regulating the NF-κB signaling pathway [20]. Studies have shown that a variety of TRIM proteins were associated with autophagy process [21–31]. However, there is no literature reporting the function of GNIP1 in autophagy at present. Here, we confirmed that GNIP1 promoted the progression of non-small cell lung cancer through inducing autophagy. We discovered that the GNIP1 protein levels were consistent with the expression of autophagy marker LC3B-II under nutrient deprivation conditions, suggesting that GNIP1 might be involved in autophagy. Further investigation verified that GNIP1 functions as a scaffold protein that recruits BECN1 and LC3B, facilitating the formation of autophagosome. Besides, GNIP1, as an E3 ubiquitin ligase, could degrade 14-3-3ζ via K48-linked ubiquitination, thus promoting Vps34 kinase activity. These results demonstrate that GNIP1 is an important participant in autophagy, which contributes to the progression of non-small cell lung cancer.

## Materials and methods

### Cell lines and culture

HEK293T, Human lung epithelial cells (BEAS-2B) and the non-small cell lung cancer cell lines (H1299, A549, H292, PC9) were purchased from the American Type Culture Collection (ATCC, Manassas, VA, USA). SPC-A1 was obtained from the National Infrastructure of Cell Line Resource (Beijing, China). The HEK293T cells were cultured in Dulbecco modified Eagle medium (DMEM) (Gibco; C11995500BT; Grand Island; NY; USA). BEAS-2B, A549, H292, PC9, SPCA1 were cultured with RPMI 1640 (Invitrogen; C11875500BT).

### Patient samples

Fresh non-small cell lung cancer tissues were obtained from the First Affiliated Hospital of Nanchang University. All patients had not received preoperative chemotherapy, radiotherapy, or immunotherapy based on histopathological criteria. The samples were immediately frozen and stored at − 80 °C for subsequent experimental analysis.

### Antibodies, reagents and plasmids

GNIP1/RNF90 (Abcam; ab170538), β-Actin (Proteintech; 66,009–1-lg), GAPDH (OriGene; TA802519), DDDDK Tag (Proteintech; 20,543-1-AP), LC3B (Proteintech; 18,725–1-AP), SQSTM1/p62 (Proteintech; TA502127), BECN1 (Proteintech; 11,306–1-AP), ULK1 (Cell Signaling Technology; 6439), HA-tag (Thermo Fisher Scientific; 26,183), GFP (Proteintech; 66,002-1-lg), UB antibody (Proteintech; 10,201–2-AP), K48 (Cell Signaling Technology; 8081), K63 (Cell Signaling Technology; 5621), PIK3C3/VPS34 (Thermo Fisher Scientific; PA5-35,215), 14–3-3ζ (Proteintech; 14,881-1-AP), anti-His (Proteintech; 10,001-0-AP/ 66,005-1-LG), anti-V5 (Proteintech; 14,440-1-AP). Cycloheximide (Sigma; C7698), DMSO (Sigma; D2650), MG132 (Biovision; 1791-5). Myc-DDDK-Tag-GNIP1, pcDNA3.1-Flag-GNIP1-W57A, PCDH-Flag-GNIP1, pEGFP-C2-LC3B, pCMV-HA-BECN1, pCMV-HA, pcDNA3.1-Flag-PIK3C3, pCMV-Flag-14-3-3ζ, pcDNA3.1-V5-BBCC, pcDNA3.1-V5-His-RBB and pcDNA3.1-V5-His-CCB plasmids were constructed by ourselves.

### Cell transfection of siRNAs and plasmids

For siRNAs knockdown, when the cell density was about 50%, we carried out the experiment according to the instructions of Pufei siRNA transfection reagent kit (Pufei, 2103–100). After 48 h, the interference effect was detected by western blot using corresponding antibody. The BECN1 siRNAs were ordered from Thermo Fisher Scientific (1,299,001).

For plasmids transfection, when the cell density was about 70–80%, we carried out the experiment according to the instructions of Pufei plasmids transfection reagent kit (Pufei, 2102–100). After 48 h, the expression effect was detected by western blot using corresponding antibody.

### Western blot and immunoprecipitation

The cells were lysed in NP-40 buffer containing PMSF. The cell lysate was centrifuged at 12,000 rpm at 4 °C for 15 min. Then cell supernatant was collected. For western blotting, an appropriate amount of 2 × loading buffer was added to denaturate the protein. SDS-PAGE gel electrophoresis was performed. After electrophoresis, the membrane (Millipore; IPVH00010) was transformed, incubate with 5% milk (BD; 232,100), subsequently, the protein was combined with primary antibody and incubated overnight. The next day, washing the membrane with 1 × TBST in a room temperature shaker three times for 10 min each time and the secondary antibodies were incubated at room temperature for 1 h. Washing three times and developing imaging (TIANGEN; PA112-01).

For immunoprecipitation, protein G agarose beads (Roche; 11,243,233,001) were added to the cell supernatant and incubate at 4 °C for 1 h. The corresponding antibodies were then added to the above supernatant containing protein G agarose beads and incubated overnight at 4 °C. Next, the immune complex was washed 3 times. Mix beads with 2 × loading buffer and boil to denaturate and carried out western blot. For the ubiquitylation assays, SDS was added in the cell lysis buffer at a final concentration of 1%, then the lysate was heated for 10 min to disrupt non-covalent interactions, and the cell extract was diluted down to 0.1% SDS before immunoprecipitating the target protein, followed by the above immunoprecipitation procedures.

### Immunohistochemistry

The tissues were fixed in neutral formalin and buried in wax. The tissue of the mouse xenograft specimen was sectioned and placed on a slide for histopathological evaluation. Briefly, tissue was dewaxed, and heated with EDTA buffer for 40 min. The slides were incubated with appropriate primary antibodies in accordance with the standard antigen retrieval protocol. The primary antibodies Ki67 (ab15580; abcam), TTF1(ab76013; abcam), LC3B (14,600-1-AP; proteintech), SQSMT1 (UMAB12; origene) were incubated overnight at 4 °C. Washing three times, the second antibody was incubated for 1 h. Add color solution to develop. ImageScope software was used to analyze tissue microarray images.

### Cell proliferation assay

3000 or 5000 cells were cultured in plates. A 24-well plate was collected every 48 h for 6 consecutive days, and the medium was replaced every 24 h. At the specified time, 4% formaldehyde was fixed at room temperature for 30 min, and 0.1% crystal violet was stained. Then, remove the crystal violet and add 10% acetic acid to dissolve. Measuring the absorbance at 595 nm and the relative growth curve was plotted.

### Transwell assay and scratch wound healing assay

Add 10^5^ cells to a chamber containing 200 ul 1% FBS medium, add 500 ul 10% FBS medium to the 24-well plate. After 12 h in an incubator at 37 °C, the chambers were taken out, then soaked in 95% ethanol for 20 min. Washing with PBS and staining with crystal violet. Finally, the cells were photographed under a microscope (Olympus; IX71).

For the scratch wound healing assay, the cells were seeded in a 6-well plate and scratched with a 200 ul pipet tips. when the density reached about 90%. Washing with PBS until no suspended cells. Cells were cultured with 1% FBS medium and photographed under a microscope (Olympus; IX71) every 12 h until the scratches healed.

### Subcutaneous xenograft assay

The 10^7^ cells were re-suspended in serum-free medium 1640 (100 μL), mixed with an appropriate amount of matrigel, and then inoculated subcutaneously into 4 week-old male BALB/ c-nu nude mice (GemPharmatech CO. Ltd, Nanjing, China) with 1 ml syringe. The mice were fed in the animal center of SPF class. The mice and tumor formation were observed every day. After one month, the mice were anesthetized and sacrificed, and the tumor volume was weighed and measured.

### Statistical analysis

Three independent replicates were performed for each experiment. All data are represented as mean ± SD. Differences in quantitative data between the two groups were analyzed using the t-test. The Kaplan–Meier method and the log-rank test were used for survival analysis. *p* < 0.05 (*), *p* < 0.01 (**), *p* < 0.001 (***) and *p* < 0.0001 (****) indicated statistically significant. Statistical analysis was performed using SPSS software version 21.0 (SPSS, Chicago, IL, USA).

## Results

### GNIP1 is upregulated in NSCLC and predicts a poor clinical outcome

**Fig. 1:**
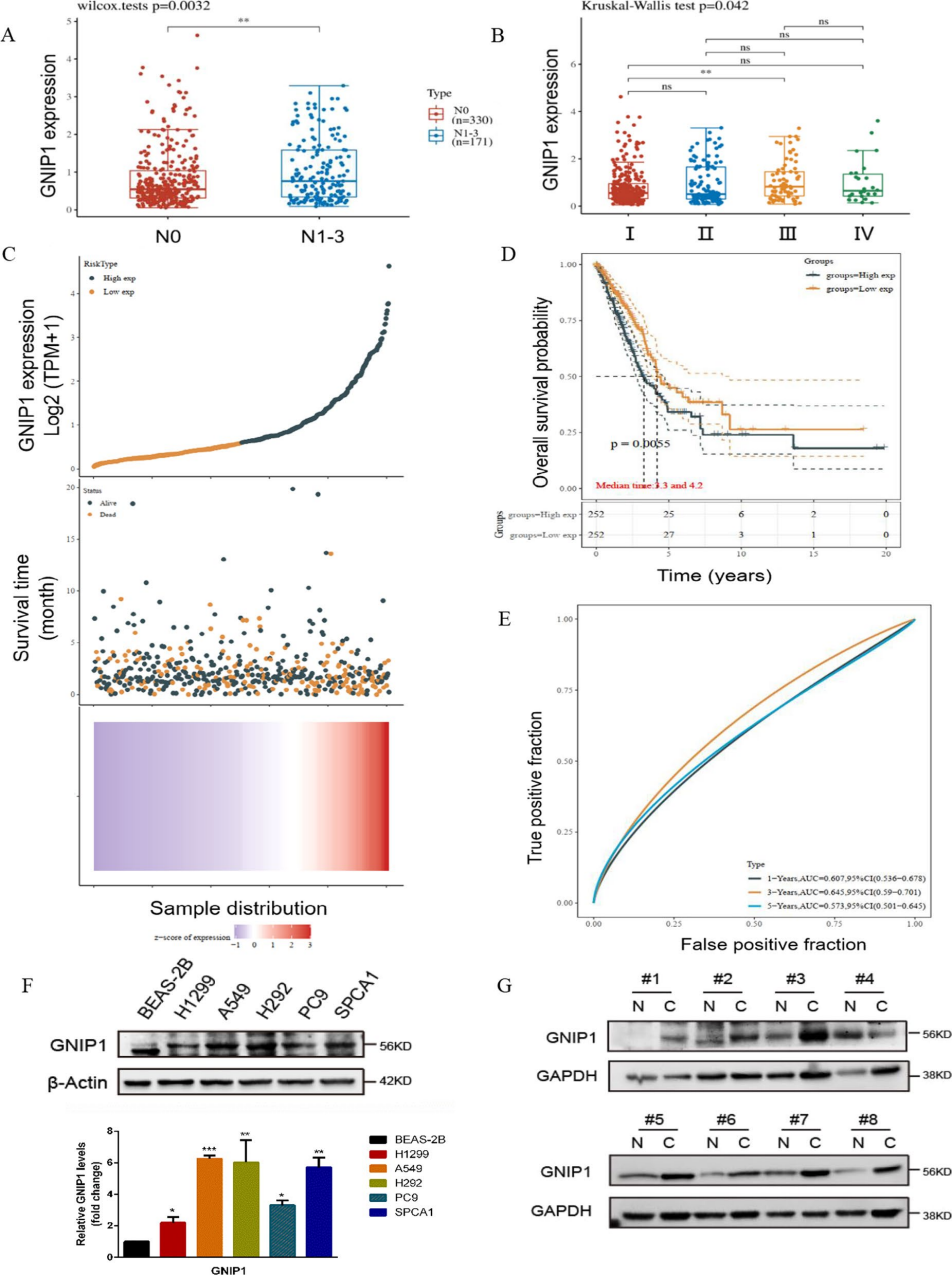
GNIP1 is highly expressed in NSCLC and predicts a poor clinical outcome. **A** The mRNA expression distribution of GNIP1 in N_0_ stage and N_1-3_ stage NSCLC tissues, N represents the stages of lymph node metastasis. **B** The mRNA expression distribution of GNIP1 in NSCLC tissues with different pathological stages. **C** Analysis of GNIP1 mRNA expression, survival time and survival status in TCGA data set. The patient samples were divided into high-expression group and low-expression group according to the median value of GNP1 mRNA expression. The upper panel was the scatter diagram of the mRNA expression of GNIP1, different colors represent different expression groups. The middle panel was the scatter diagram distribution of survival time and survival state corresponding to the expression of different sample GNIP1. The lower panel represents a heat map of the GNIP1 mRNA expression (All X-axis represents the NSCLC patient samples distribution). The GNIP1 expression analysis was produced by pheatmap package (R version, 3.6.2, pheatmap version, 1.0.8). **D** The relationship between the mRNA expression of GNIP1 and the possibility of survival in NSCLC patients. **E** Receiver operating characteristic curve analysis of GNIP1 at different time, in which the higher the AUC value, the stronger the predictive ability of survival conditions. AUC, area under the curve. **F** Western blot to check the protein levels of GNIP1 in NSCLC cells and normal lung epithelial cells (BEAS-2B) (upper panel). Quantitative gray value analysis of GNIP1 expression (lower panel). Data are presented as the average of three independent experiments (mean ± SD). *, *p* < 0.05, **, *p* < 0.01, ***, *p* < 0.001. **G** Western blot detected the expression of GNIP1 in tissues of 8 patients with non-small cell lung cancer. C: cancer tissues; N: normal tissues

To identify whether GNIP1 is related to progression of NSCLC, we performed a series of bioinformatics analyses using the TCGA database (http://cancergenome.nih.gov/). First, the relationship between GNIP1 mRNA expression level and clinicopathological features of patients with NSCLC was analyzed. The mRNA expression of GNIP1 in N_1_-N_3_ stage was higher than that in N_0_ stage (Fig. 1A), and the mRNA expression of GNIP1 in stage III was higher than that in stage I (Fig. 1B). Then, patients were divided into high-expression group and low-expression group according to the median value of GNP1 mRNA expression. Distribution of survival status revealed that patients in the high-expression group had higher mortality and a shorter survival time than those in the low-expression group (Fig. 1C), and Kaplan–Meier survival curve confirmed that the high expression of GNIP1 can shorten the survival time (Fig. 1D). In addition, the prediction capability of the GNIP1 was further analyzed using ROC curves, the expression of GNIP1 can accurately predict the 1-, 3-, or 5-year survival rate of NSCLC patients, and the 3-year survival rate has the highest accuracy (AUC = 0.645) (Fig. 1E). To further investigate the crucial role of GNIP1 in non-small cell lung cancer, we evaluated the relationship between GNIP1 expression and clinicopathological characteristics of NSCLC patient (Table 1). GNIP1 expression levels were closely related to lymph node metastasis stage and pathological stage (*p < 0.018 and *p < 0.019, respectively). These results suggested that GNIP1 was related to the progression of NSCLC.

**Table 1:** Association of GNIP1 expression levels with clinicopathologic characteristics in NSCLC

Clinicopathologic	Levels	GNIP1 expression		*p*-value
Characteristics		Low expression group	High expression of group	
*n*		267	268	0.158
Age, *n* (%)	≤ 65	137 (51.3%)	118 (44.0%)	
	> 65	123 (46.1%)	138 (51.5%)	
	Unknown	7 (2.6%)	12 (4.5%)	
Race, *n* (%)	Asian	2 (0.7%)	5 (1.9%)	0.479
	Black or African American	26 (9.7%)	29 (10.8%)	
	White	205 (76.8%)	201 (75.0%)	
	Unknown	34 (12.7%)	33 (12.3%)	
Gender, *n* (%)	Female	154 (57.7%)	132 (49.3%)	0.062
	Male	113 (42.3%)	136 (50.7%)	
	Unknown	0	0	
Smoker, *n* (%)	No	41 (15.4%)	34 (12.7%)	0.465
	Yes	220 (82.4%)	226 (84.3%)	
	Unknown	6 (2.2%)	8 (3.0%)	
T stage, *n* (%)	T1	90 (33.7%)	85 (31.7%)	0.636
	T2	147 (55.1%)	142 (53.0%)	
	T3	21 (7.9%)	28 (10.4%)	
	T4	8 (3.0%)	11 (4.1%)	
	Unknown	1 (0.3%)	2 (0.7%)	
N stage, *n* (%)	N0	188 (70.4%)	160 (59.7%)	0.018*
	N1	42 (15.7%)	53 (19.8%)	
	N2	27 (10.1%)	47 (17.5%)	
	N3	1 (0.4%)	1 (0.4%)	
	Unknown	9 (3.4%)	7 (2.6%)	
M stage, *n* (%)	M0	194 (72.7%)	167 (62.3%)	0.461
	M1	11 (4.1%)	14 (5.2%)	
	Unknown	62 (23.2%)	87 (32.5%)	
Pathologic stage, *n* (%)	Stage I	157 (58.8%)	137 (51.1%)	0.019*
	Stage II	65 (24.3%)	58 (21.6%)	
	Stage III	29 (10.9%)	55 (20.5%)	
	Stage IV	12 (4.5%)	14 (5.2%)	
	Unknown	4 (1.5%)	4 (1.5%)	
Primary therapy outcome, *n* (%)	PD	36 (13.5%)	35 (13.1%)	0.959
	SD	17 (6.4%)	20 (7.5%)	
	PR	3 (1.1%)	3 (1.1%)	
	CR	169 (63.3%)	163 (60.8%)	
	Unknown	42 (15.7%)	47 (17.5%)	

*p*-value were calculated by comparing the high expression group and the low expression group of GNIP1, using a Chi-square test. *p* < 0.05 was considered statistically significant. * *p* < 0.05, **p < 0.01, *** *p* < 0.001

Our previous study showed that TRIM7, the shortest form of *TRIM7* gene, affects cancer progression through regulating NF-κB signaling pathway [20]. However, the function of GNIP1 has not been fully reported. We next checked the protein level of GNIP1 in NSCLC cells and tissues. Comparing with *TRIM7,* we found an interesting phenomenon that compared with normal human lung epithelial cells (BEAS-2B), the protein level of GNIP1 was obviously upregulated in NSCLC cells, and the expression of GNIP1 in non-small cell lung cancer tissues was increased (Fig. 1F, G). Taken together, these results demonstrated that GNIP1 was up-regulated in NSCLC and may contribute to NSCLC progression.

### GNIP1 promotes the proliferation and migration of NSCLC cells

**Fig. 2:**
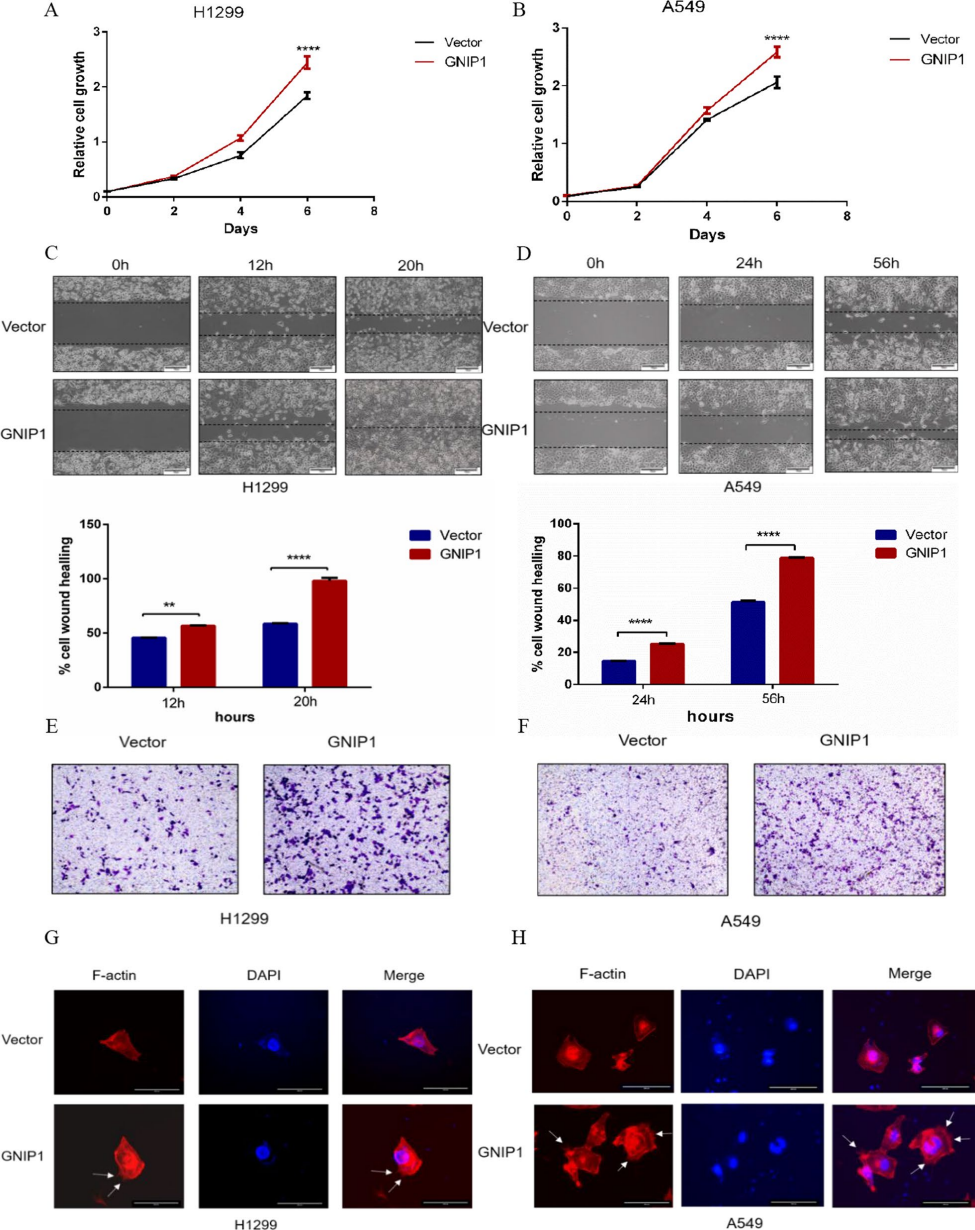
GNIP1 promotes the proliferation and migration of NSCLC cells. **A**, **B** Cell counting experiment detected the growth of H1299 and A549 cells after overexpression of GNIP1. Data are presented as the average of three independent experiments (mean ± SD). ****, *p* < 0.0001. **C**, **D** The scratch wound healing assays of H1299 and A549 cells with or without exogenous GNIP1 expression (upper panel). Statistical analysis of cell scratch wound healing assays between groups (lower panel). Data are presented as the average of three independent experiments (mean ± SD). **, *p* < 0.01, ****, *p* < 0.0001 **E**, **F** Transwell assays of H1299 and A549 cells with or without ectopic GNIP1 expression. **G**, **H** F-actin staining assays of H1299 and A549 cells with or without exogenous GNIP1 expression

Given that GNIP1 may be a potential oncogene, we investigated the role of GNIP1 in NSCLC. Cell crystal violet was performed with H1299 and A549 cells. Vector plasmid and Flag-GNIP1 plasmid were transfected into the cells to detect cell proliferation. The results showed that overexpressing GNIP1 promoted the growth of H1299 (Fig. 2A) and A549 cells (Fig. 2B). Next, we evaluated the role of GNIP1 in NSCLC cell migration. The wound healing assays showed that overexpression of GNIP1 in H1299 and A549 cells significantly increased the migration rate (Fig. 2C, D). To further demonstrate this effect, the transwell assays were performed. The results showed that compared to the control group, the overexpression of GNIP1 in H1299 and A549 cells increased the number of migrated cells (Fig. 2E, F). Furthermore, when GNIP1 was overexpressed in H1299 and A549 cells, the number of pseudopodia increased in F-actin staining experiment (Fig. 2G, H). The overexpression efficiency of GNIP1 in H1299 and A549 cells was detected by Western blot (Additional file 1: Fig. S1A, B). Altogether, the above results indicated that GNIP1 facilitated the proliferation and migration of NSCLC cells.

### GNIP1 recruits autophagy-related proteins LC3B and BECN1 to induce autophagy

**Fig. 3:**
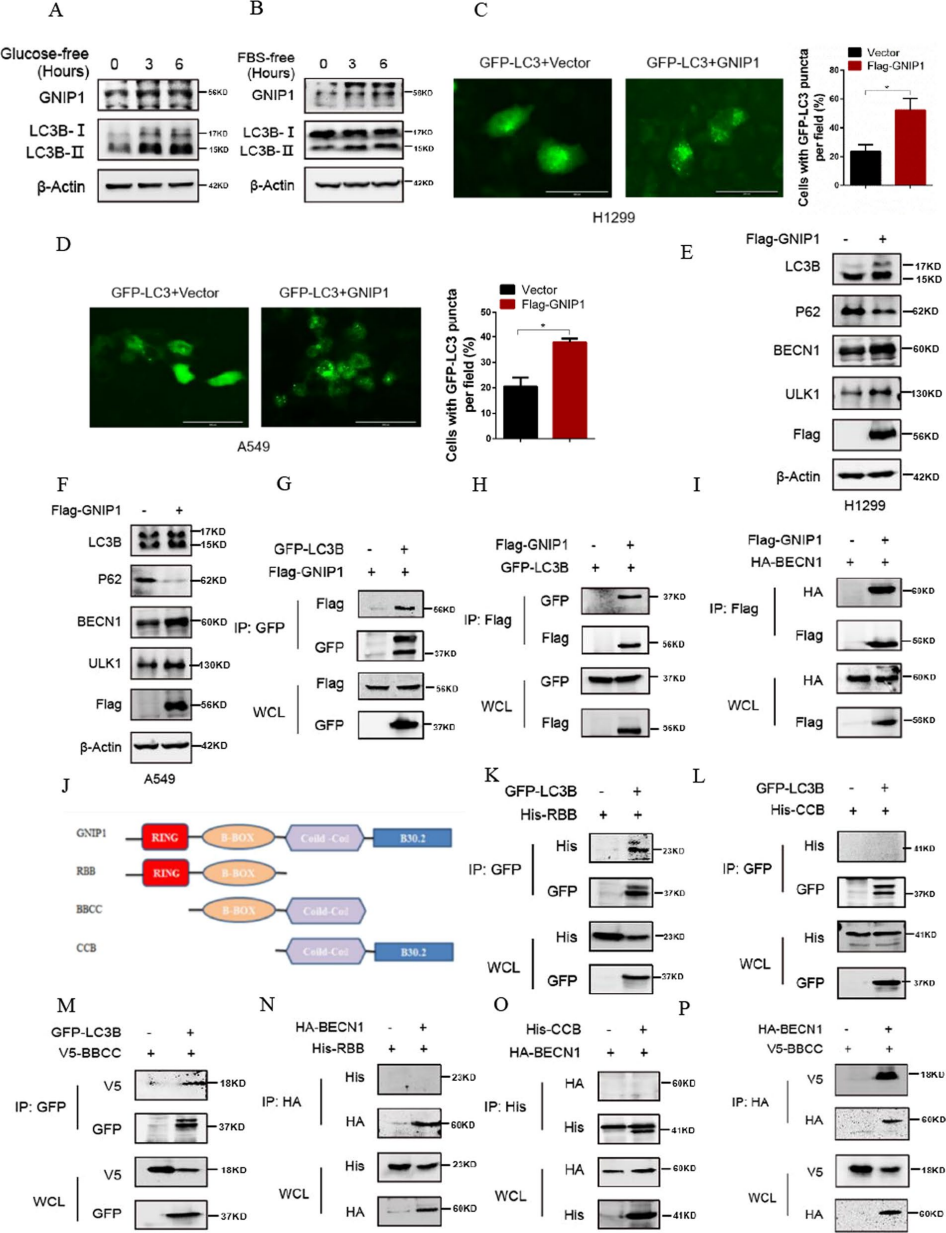
GNIP1 induces autophagy by recruiting LC3B and BECN1. **A**, **B** Protein expression levels of GNIP1 and LC3B in A549 cells treated with glucose- or serum-free medium as determined by western blot analysis. **C**, **D** Fluorescence microscopy was used to detect the number of autophagosomes in H1299 and A549 cells stably expressing LC3B after overexpressing GNIP1 (Olympus IX83) (left panel). Statistical analysis of the number of GFP puncta between groups under 200 × magnification. Data are presented as the average of three independent experiments (mean ± SD). *, *p* < 0.05 (right panel). **E**, **F** The expression level of autophagy related proteins was detected after overexpression of GNIP1 in H1299 and A549 cells. **G**, **H** Co-Immunoprecipitation assays of GNIP1 and LC3B. **I** Co-Immunoprecipitation assays of GNIP1 and BECN1. **J** Structural diagram of GNIP1 mutant. K-M LC3B and GNIP1 binding domain, immunoprecipitation assays. H1299 cells transfected with His-RBB and GFP-LC3B plasmids **K**, His-CBB and GFP-LC3B plasmids **L** or V5-BBCC and GFP-LC3B plasmids **M** were subjected to a Co-IP assay. N-P BECN1 and GNIP1 binding domain, immunoprecipitation assays. H1299 cells transfected with His-RBB and HA-BECN1 plasmids **N**, HA-BECN1 and His-CBB plasmids **O** or V5-BBCC and HA-BECN1 plasmids **P** were subjected to a Co-IP assay

Based on the analysis of primary structure of the conserved protein, GNIP1, the longest isoform, has an NH_2_-terminal RING finger domain, a B-box domain, a coiled-coli domain, and a B30.2 domain at the end of the COOH terminal, indicating that GNIP1 may be involved in multiple biological processes. Studies have shown that many TRIM family proteins are involved in autophagy [32]. However, studies on GNIP1 are still focused on E3 ubiquitin ligase activity and glycogen metabolism. Whether GNIP1 participates in autophagy has not been reported. We discovered that the expression of GNIP1 was up-regulated in A549 cells treated with glucose-free or serum-free medium, which was in accordance with the expression of LC3B-II (Fig. 3A, B). We then examined the effects of GNIP1 on autophagy induction from morphology in NSCLC cells according to the guidelines [33]. To observe the number of autophagosomes, we used LC3B plasmid with green fluorescence to construct H1299 and A549 stable cell line with LC3B overexpression. As shown in Fig. 3C, D, GNIP1 overexpression increased the number of GFP-LC3B autophagosomes. To further validate this phenomenon, autophagy-related proteins (LC3B, SQSTM1/p62, BECN1 and ULK1) were detected to explore the relationship between GNIP1 and autophagy. As expected, overexpression of GNIP1 increased the expression of LC3B-II, BECN1 and total ULK1, while decreased the expression of SQSTM1/P62 in H1299-LC3B stable cells (Fig. 3E) and A549 cells (Fig. 3F). These results indicated that overexpression of GNIP1 could induce autophagy in NSCLC.

The above studies suggested that the expression of autophagy-related proteins might have a certain relationship with GNIP1. Because GNIP1 contains domains that bind to proteins and regulate protein homeostasis, we speculated that GNIP1 might act as a scaffold protein and played a regulatory role in autophagy by recruiting autophagy-related proteins such as LC3B and BECN1. To verify this hypothesis, coimmunoprecipitation (Co-IP) assays were conducted to detect the interaction of GNIP1 with LC3B and BECN1. Figure 3G–I showed that GNIP1 could bind to LC3B and BECN1 in H1299 cells. Together with the data above, these results indicated that GNIP1, as a scaffold protein, recruited LC3B and BECN1 to participate in the regulation of autophagy.

To further clarify the exact binding sites of GNIP1 with LC3B and BECN1, we conducted a series of truncated plasmids of GNIP1. GNIP1 contains four domains: the RING domain, B-box domain, Coiled-coil domain and B30.2 domain [12]. Therefore, we conducted three truncated plasmids, including RBB mutant containing RING and B-box domains, BBCC mutant containing B-box and Coiled-coil domains, and CCB mutant containing Coiled-coil and B30.2 domains (Fig. 3J). In H1299 cells, LC3B plasmids were co-transfected with RBB, CCB and BBCC mutants respectively, and then Co-IP assays were performed. The results showed that RBB and BBCC mutants specifically bound to LC3B (Fig. 3K–M), while CCB mutants did not bind to LC3B (Fig. 3L). These results demonstrated that the binding site between GNIP1 and LC3B was located in the B-box domain. Using the similar experimental method, BECN1 plasmids were co-transfected with RBB, CCB and BBCC mutants in H1299 cells respectively, the Co-IP assays showed that the binding site of GNIP1 and BECN1 was located in the B-box and Coiled-coil domains, indicating that the specific binding of BECN1 and GNIP1 required the co-existence of B-box and Coiled-coil domains (Fig. 3N–P). These results suggested that GNIP1 induced autophagy by interacting with LC3B and BECN1 in NSCLC.

### GNIP1 regulates the protein homeostasis of 14–3-3ζ by ubiquitination

**Fig. 4:**
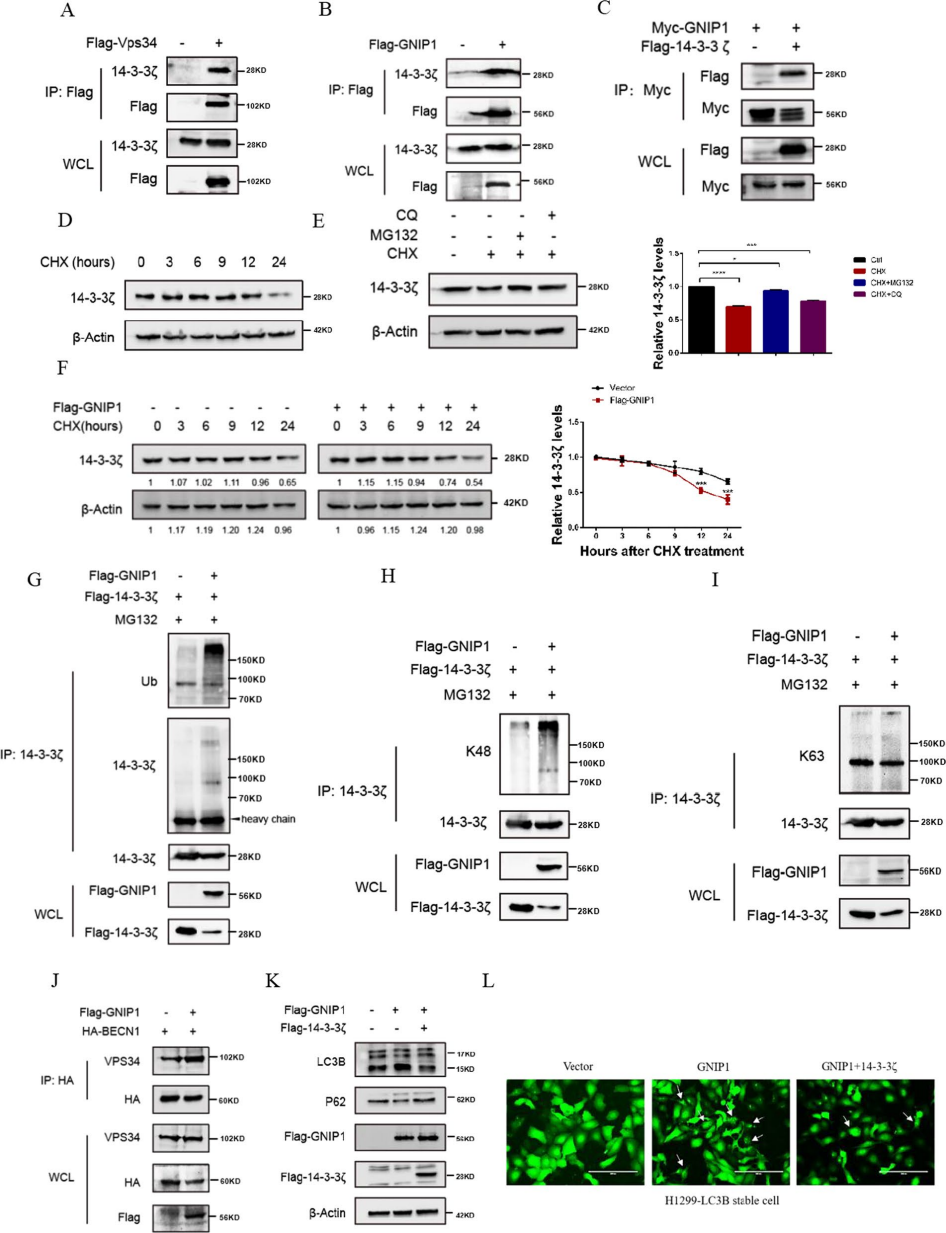
GNIP1 promotes the degradation of 14-3-3ζ by ubiquitination. **A** Interaction of Vps34 with 14-3-3ζ in H1299 cells. **B** Co-Immunoprecipitation assays of GNIP1 and 14-3-3ζ. **C** An exogenous interaction GNIP1 with 14-3-3ζ in H1299 cells. **D** Western blot was used to detect the degradation rate of 14-3-3ζ after CHX treatment in A549 cells. **E** 25 μg/ml CHX, 10 μM MG132 or 20 μM CQ were used to infect A549 cells for 12 h, and then performed western blot to detect the protein level of 14-3-3ζ (left panel). Quantitative statistical analysis of 14-3-3ζ protein (right panel). Data are presented as the average of three independent experiments (mean ± SD). *, *p* < 0.05, ***, *p* < 0.001, ****, *p* < 0.0001. **F** GNIP1 plasmids were transfected in A549 cells, the degradation rate of 14-3-3ζ was checked by western blot (left panel). Quantitative statistical degradation curve of 14-3-3ζ protein (right panel). Data are presented as the average of three independent experiments (mean ± SD). ***, *p* < 0.001. **G** Overexpressing GNIP1 in H1299, and treated with 10 μM MG132 for 12 h, the ubiquitination level of 14-3-3ζ was checked by western blot. **H**, **I** Overexpressing GNIP1 in H1299, and treated with 10 μM MG132 for 12 h, the K48-and K63-ubiquitination levels of 14-3-3ζ were analyzed by Western blot **J** Ectopic GNIP1 increases BECN1-VPS34 interactions. **K** H1299-LC3 stable cells were co-transfected with Flag-GNIP1 and Flag-14-3-3ζ plasmids. The expression of LC3B and P62 were detected by western blot. **L** The number of autophagosomes in H1299 cells after overexpression of GNIP1 or 14-3-3ζ was observed by fluorescence microscope

Next, we further explored the regulatory mechanism of GNIP1 involved in autophagy. 14-3-3ζ, also known as YWHAZ, is an important member of the highly conserved 14-3-3 family and plays a key role in many biological processes in eukaryotic cells [34–36]. 14-3-3ζ has been shown to be an important negative regulator of autophagy, which directly interacts with Vps34 to form a complex, thus blocking autophagy [11, 35, 37]. We demonstrated the interaction between 14-3-3ζ and Vps34 in H1299 cells (Fig. 4A). Interestingly, we found that GNIP1 also interacted with 14-3-3ζ in H1299 cells (Fig. 4B, C). As GNIP1 is an E3 ubiquitin ligase, we want to clarify if GNIP1 could influence the ubiquitination of 14-3-3ζ. We analyzed the stability of 14-3-3ζ by treating H1299 cells with cycloheximide (CHX). After CHX treatment, we found that 14-3-3 ζ protein levels were reduced at 24 h (Fig. 4D) and this reduction could be blocked by MG132, but not CQ (chloroquine) (Fig. 4E). The above results suggested that degradation of 14-3-3ζ occurs in the proteasome pathway. To check if GNIP1 regulated the stability of 14-3-3ζ, GNIP1 was overexpressed in H1299 cells, and the protein level of 14-3-3ζ was detected under the treatment of CHX. Interestingly, exogenous expression of GNIP1 made the protein level of 14-3-3 ζ decrease rapidly from the 9th hour (Fig. 4F). These results showed that GNIP1 affected the stability of 14-3-3ζ.

We next detected whether GNIP1 affected the ubiquitination of 14-3-3ζ. As expected, the ubiquitination level of 14-3-3ζ was increased when overexpressing GNIP1 (Fig. 4G). Ubiquitination is an important post-transcriptional modification and different types of ubiquitin chains can be recognized by different ubiquitin binding proteins and play different roles [38]. K48- and K63-linked ubiquitination are classical types of ubiquitination, the K48-linked ubiquitination is usually a protein degradation signal, and its substrate protein will enter the proteasome for degradation, however, the K63-linked ubiquitination is often related to the activation or translocation of proteins, and its substrate protein may be degraded by lysosomal endocytosis [39]. We detected the ubiquitination type of 14-3-3ζ mediated by GNIP1. We found that the overexpression of GNIP1 elevated the K48-linked ubiquitination, but had no effect on the K63-linked ubiquitination of 14-3-3ζ (Fig. 4H, I). The above results indicated that GNIP1 promoted the degradation of 14-3-3ζ by mediating K48-linked ubiquitination.

It has been reported that BECN1-PIK3C3/Vps34-PIK3R4 complex plays an essential role in the formation of autophagosome membrane structure [40–43]. Next, we transfected GNIP1 plasmid in H1299 cells to detect whether the binding strength between BECN1 and Vps34 was increased. Co-IP assay results showed that overexpression of GNIP1 could increase the interaction between BECN1and Vps34 (Fig. 4J). We further investigated whether GNIP1 promoted autophagy by mediating 14-3-3ζ ubiquitination. Exogenous GNIP1 and 14-3-3ζ plasmids were transfected into H1299-LC3B stable cells, then western blot assays showed that overexpressing GNIP1 increased LC3B-II level and decreased SQSMT2 (P62) protein level, while 14-3-3ζ overexpression inhibited this effect induced by GNIP1 (Fig. 4K). Fluorescence experiments further confirmed this result. The number of autophagosomes increased when overexpressing GNIP1 and 14-3-3ζ overexpression decreased the autophagosomes (Fig. 4L). In summary, the results indicated that GNIP1 promoted autophagy via ubiquitinating and degrading 14-3-3ζ.

### The ubiquitination of 14–3-3 ζ relies on the E3 ligase activity of GNIP1

**Fig. 5:**
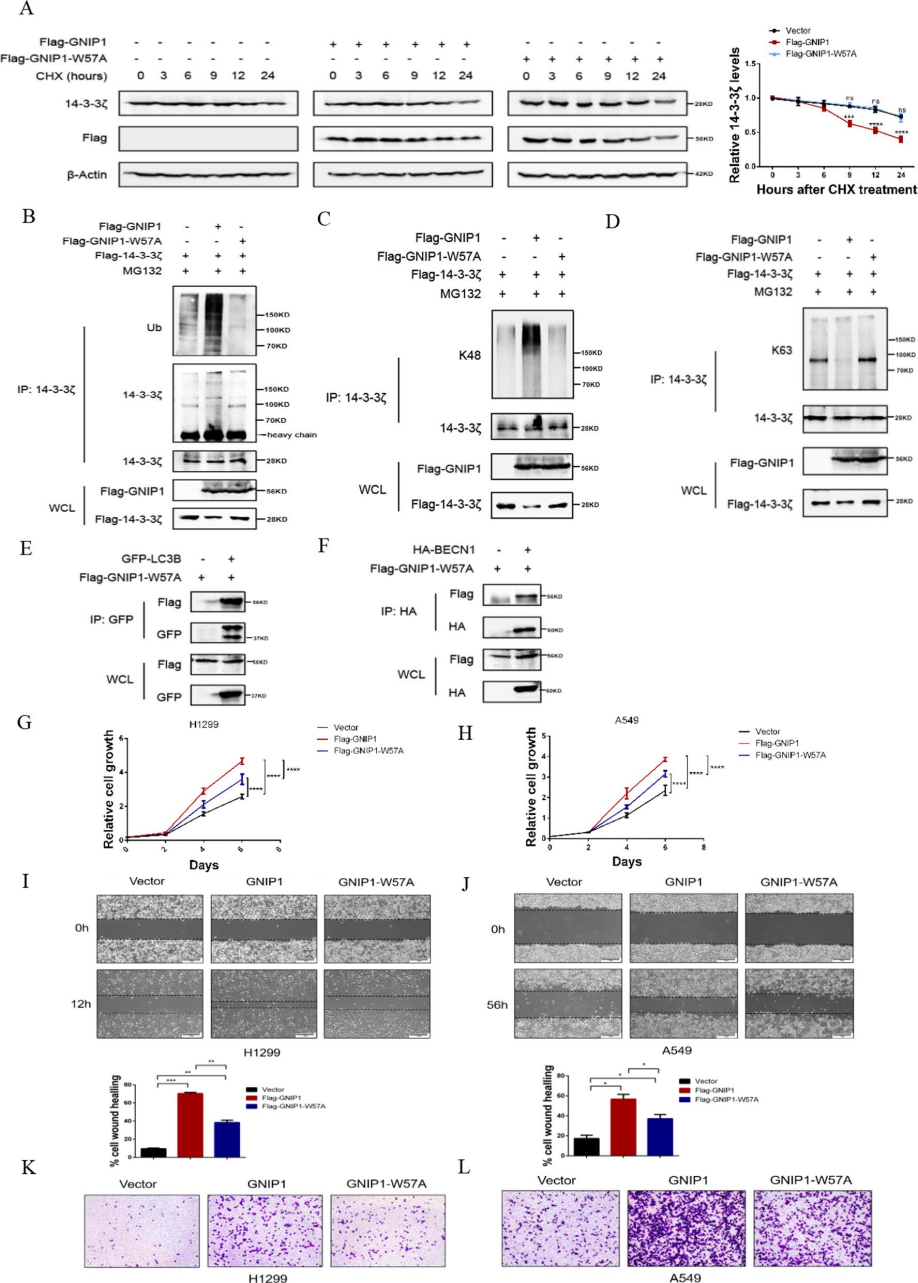
The ubiquitination of 14-3-3 ζ relies on the E3 ligase activity of GNIP1 **A** GNIP1or GNIP1-W57A plasmids were transfected in A549 cells, the degradation rate of 14-3-3ζ was checked after CHX treatment by western blot (left panel). Quantitative statistical degradation curve of 14-3-3ζ protein (right panel). Data are presented as the average of three independent experiments (mean ± SD). ***, *p* < 0.001, ****, *p* < 0.0001, ns, no significance. **B** 14-3-3ζ plasmids and GNIP1or GNIP1-W57A plasmids were transfected in H1299 cells, and treated with 10 μM MG132 for 12 h, the ubiquitination level of 14-3-3ζ was checked by western blot. **C**, **D** Overexpressing 14-3-3ζ plasmids and GNIP1or GNIP1-W57A plasmids in H1299, and treated with 10 μM MG132 for 12 h, the K48-and K63-ubiquitination levels of 14-3-3ζ were analyzed by Western blot. **E** Interaction of GNIP1-W57A with GFP-LC3B in H1299 cells. **F** Interaction of GNIP1-W57A with HA-BECN1 in H1299 cells. **G**, **H** GNIP1 or GNIP1-W57A plasmids were transfected in H1299 and A549 cells, the cell growth ability was detected. Data are presented as the average of three independent experiments (mean ± SD). ****, *p* < 0.0001. **I**, **J** The scratch wound healing assays of H1299 and A549 cells with exogenous GNIP1 or GNIP1-W57A expression (upper panel). Statistical analysis of cell wound healing assays between groups (lower panel). Data are presented as the average of three independent experiments (mean ± SD). *, *p* < 0.05. **, *p* < 0.01, ***, *p* < 0.001. **K**, **L** Transwell assays of H1299 and A549 cells with exogenous GNIP1 or GNIP1-W57A expression

To further confirm the E3 ligase activity of GNIP1 is necessary for 14-3-3ζ ubiquitination, we mutated tryptophan residue to alanine and constructed an E3 ligase-inactive mutant (GNIP1-W57A), which blocks the binding of RING domain to ubiquitin- conjugating enzymes [17]. The effect of GNIP1-W57A on the stability of 14-3-3ζ was examined, the results showed that overexpression of GNIP1 accelerated 14-3-3ζ degradation, while ligase-inactive mutant W57A did not (Fig. 5A). Besides, in vivo ubiquitination experiments demonstrated that GNIP1 promoted the ubiquitination of 14-3-3ζ through the K48-linked chains, while GNIP1-W57A failed to ubiquitinate 14-3-3ζ (Fig. 5B–D). Figure 3 has proved that GNIP1 also interacted with autophagy proteins to induce autophagy, we next examined whether the E3 ligase activity of GNIP1 affected its function as autophagy scaffold protein. The co-immunoprecipitation (Co-IP) assays showed that GNIP1-W57A could still interact with LC3B and BECN1 (Fig. 5E, F), indicating that GNIP1-W57A did not affect its function of scaffold protein. We investigated the effects of ligase-inactive GNIP1 mutant on the growth and migration of NSCLC cells. Cell proliferation experiments showed that both the wild-type and mutant GNIP1 could increase the proliferation of NSCLC cells. However, compared with GNIP1, GNIP1-W57A showed a relatively weak effects on the growth ability of H1299 and A549 cells (Fig. 5G, H). Wound healing and transwell assays also demonstrated consistent results that both the wild-type and mutant GNIP1 promoted cell migration, but the migration-promoting ability of mutant GNIP1 was relatively weak (Fig. 5I–L). The overexpression efficiency of GNIP1 and GNIP1-W57A in H1299 and A549 cells were detected by western blot (Additional file 1: Fig. S2A, B). Taken together, these results suggested that GNIP1 promoted autophagy by functioning both as a scaffold protein and an E3 ubiquitin ligase to enhance the proliferation and migration of NSCLC cells.

### Inhibition of autophagy attenuates the tumor-promoting effects of GNIP1

**Fig. 6:**
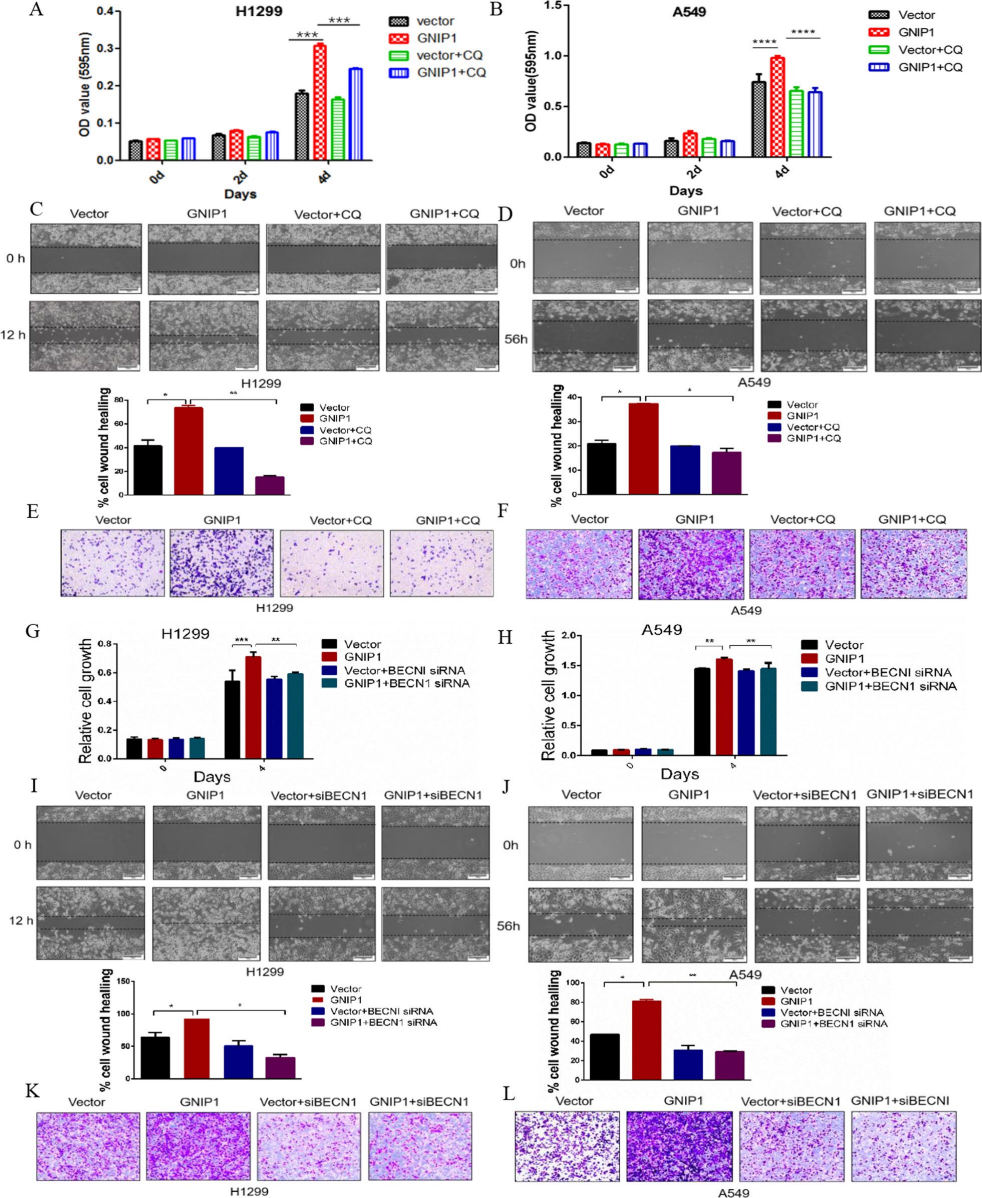
Inhibition of autophagy attenuates the tumor-promoting effects of GNIP1. A, B Cell proliferation assay. GNIP1 overexpressed in H1299 **A** and A549 cells **B** and then infected with 20 μM CQ for 12 h to performed cell counting experiment. Data are presented as the average of three independent experiments (mean ± SD). ***, *p* < 0.001, ****, *p* < 0.0001. **C**, **D** GNIP1 was overexpressed in H1299 (**C**) and A549 (**D**) cells and then infected with 20 μM CQ for 12 h for scratch test (upper panel). Statistical analysis of cell wound healing assays between groups (lower panel). Data are presented as the average of three independent experiments (mean ± SD). *, *p* < 0.05, **, *p* < 0.01. **E**, **F** GNIP1 overexpressed in H1299 (**E**) and A549 cells (**F**) and then infected with 20 μM CQ for 12 h to perform transwell migration assay. **G**, **H** Cell proliferation assay. H1299 cells (**G**) and A549 cells (**H**) were transfected with empty vector and GNIP1 plasmid or siRNA ctrl and siRNA BECN1 to performed cell counting experiment. Data are presented as the average of three independent experiments (mean ± SD). **, *p* < 0.01, ***, *p* < 0.001. **I**, **J** GNIP1 plasmid and BECN1 siRNAs were transfected into H1299 (**I**) and A549 cells (**J**) and then the scratch test was conducted (upper panel). Statistical analysis of cell wound healing assays between groups (lower panel). Data are presented as the average of three independent experiments (mean ± SD). *, *p* < 0.05, **, *p* < 0.01. **K**, **L** Transwell assay. GNIP1 plasmid and BECN1 siRNAs were transfected into H1299 (**K**) and A549 cells (**L**) and then the transwell assay was conducted

Our previous data has shown that GNIP1 contributed to the proliferation and migration of NSCLC cells (Fig. 2). We hypothesized that GNIP1 may play a role in the cancer progression through mediating autophagy. In order to demonstrate this, we infected the NSCLC cells with autophagy inhibitor chloroquine (CQ). Figure 6A, B showed that CQ treatment decreased the proliferation rate of the cells overexpressing GNIP1. Cell migration assays showed that overexpression of GNIP1 promoted cell migration in H1299 and A549 cells, but the ability of migration was significantly decreased when NSCLC cells were treated with CQ (Fig. 6C–F). The efficiency of GNIP1 overexpression and CQ inhibition were detected by western blot (Additional file 1: Fig. S3A, B). The above results confirmed that the autophagy inhibitor CQ could inhibit the effects of GNIP1 on promoting the proliferation and migration of NSCLC cells.

In order to further prove that GNIP1 promotes the proliferation and migration of NSCLC cells via autophagy, BECN1 was knocked down using siRNAs. Knocking down BECN1 significantly decreased the tumor promoting effects of GNIP1 no matter on cell proliferation or migration (Fig. 6G–L). The efficiency of GNIP1 overexpression and BECN1 siRNAs knockdown were detected by western blot (Additional file 1: Fig. S3C, D). From these results, we concluded that GNIP promoted the proliferation and migration of NSCLC cells through inducing autophagy.

### *GNIP1 increases tumour growth *in vivo

**Fig. 7:**
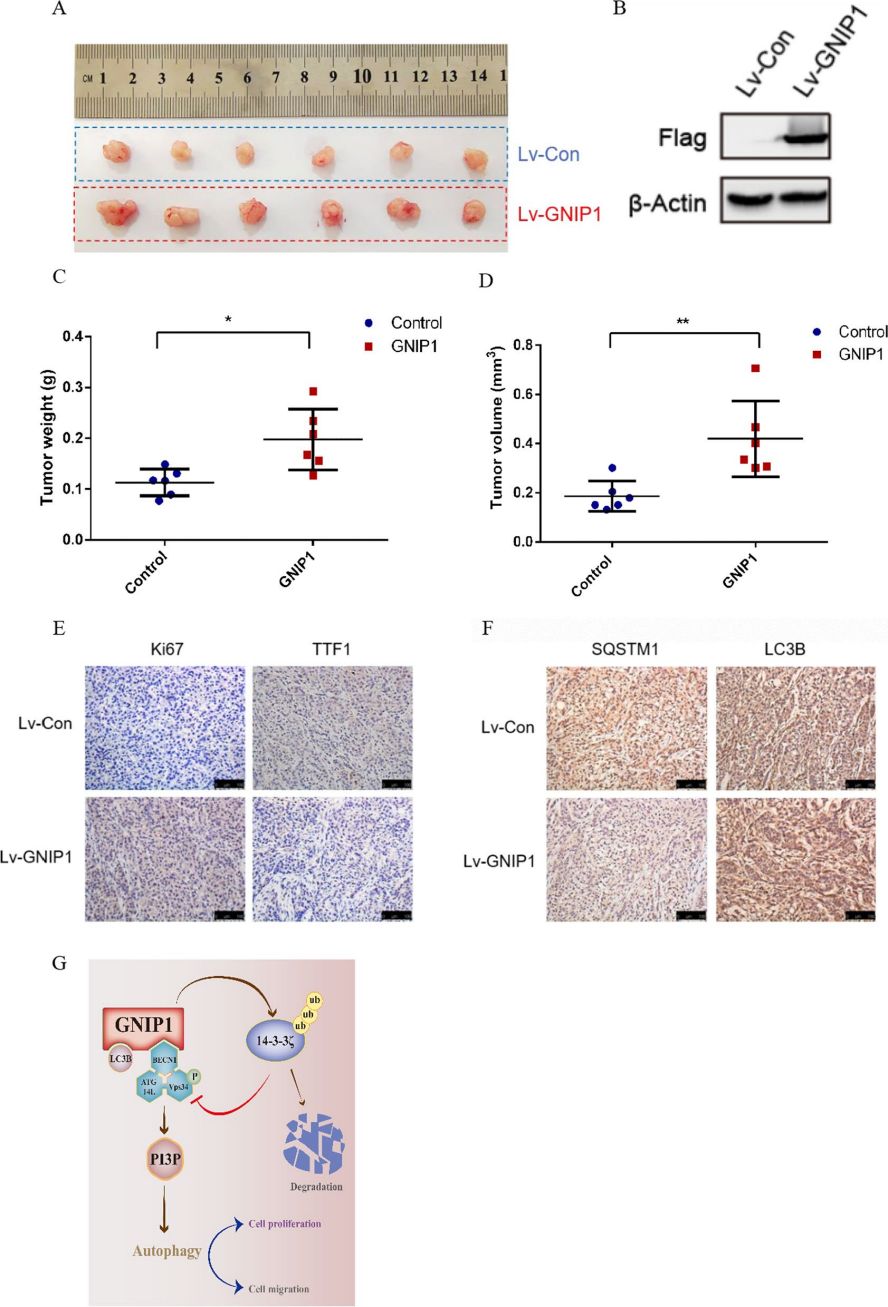
GNIP1 increases tumour growth in vivo. **A** Subcutaneous tumorigenesis assay. **B** Detection of GNIP1 overexpression effect in A549 stable cell line. **C**, **D** Tumour weights and sizes were monitored. The *p* value was calculated by paired t-test (mean ± SD, *n* = 6). *, *p* < 0.05, **, *p* < 0.01. **E**, **F** Immunohistochemistry to detect the protein levels of Ki67, TTF1, SQSTM1 or LC3B in tumours. **G** Working model of GNIP1

To further verify the function of GNIP1 in NSCLC cells, we used A549 cells with stable expression of GNIP1 to construct a xenograft model to determine whether GNIP1 has an effect on the proliferation of NSCLC cells in vivo. The results showed that GNIP1 expression led to rapid tumour growth (Fig. 7A, B), both tumor weight (Fig. 7C) and tumor volume (Fig. 7D) increased significantly. Subsequently, we performed immunohistochemical (IHC) assays, the results confirmed that in GNIP1-overexpressing tumors, the expression level of Ki67 was increased while that of TTF1 was decreased, indicating that GNIP1 can promote cell proliferation and tumour growth in vivo (Fig. 7E). In addition, to further clarify the relationship between GNIP1 and autophagy, immunohistochemistry was used to detect the expression of autophagy markers. The result showed that SQSTM1-positive cells were reduced and LC3B-positive cells were elevated in GNIP1-expressing tumours (Fig. 7F), revealing that GNIP1 is involved in autophagy.

## Discussion

Autophagy is a homeostasis mechanism in eukaryotes, cells using this biological process to meet their metabolic needs, or remove toxic aggregates from the cytoplasm to maintain homeostasis. Autophagy involves many proteins. ULK1 regulates the initiation of autophagy, and BECN1 plays a key role in the formation of autophagosome membranes [44]. The subsequent stages of the pathway are controlled by the mammalian paralogues of the yeast Atg8. Among them, LC3B is the most commonly autophagosome marker. SQSTM1/P62 is an autophagy receptor, which is degraded by lysosomes after delivery to the substrate. These autophagic proteins need to form large complexes to complete the autophagy process.

GNIP1 is a member of the TRIM family proteins, the TRIM protein usually consists of RING domain, B-box domain, and coiled-coil domain. A lot of TRIMs have variable C-terminal domains for binding substrates. TRIM family proteins are involved in a wide range of biological processes, and their alterations are associated with a variety of pathological conditions, such as neurodegenerative diseases, cancer, developmental disorders, and viral infections [45–47]. Studies have shown that TRIMs are involved in autophagy, facilitating the formation of autophagic protein complexes. The reported studies showed that B30.2/SPRY might be the key domain for assembling autophagic protein [25]. Our previous studies showed that TRIM59 participates in autophagy [48], indicating that TRIM proteins play an important role in autophagy and the function of TRIM proteins need further clarified. However, whether GNIP1 is involved in autophagy biological process is still unclear.

14-3-3 family proteins, a family of phosphoserine/threonine proteins, are involved in a variety of cellular processes, including migration, cell cycle progression, differentiation and apoptosis [49]. Seven mammalian 14-3-3 isoforms (β, γ, ε, ζ, η, σ and τ) have been reported [50]. 14-3-3ζ, one of the seven isoforms, has been reported to exert a negative regulatory role in autophagy through inhibiting hVps34 kinase activity. Currently, 14-3-3ζ degradation has not been reported. We discovered that GNIP1 interacts with 14-3-3ζ and stimulates the degradation of 14-3-3ζ through mediating K48- linked ubiquitination, thus alleviating its inhibition on autophagy.

In conclusion, our studies reveal a novel autophagy regulator-GNIP1 (Fig. 7G). Biochemically, GNIP1 could bind to BECN1 and LC3B through different domains and induce autophagy via promoting the formation of autophagic protein complexes. Besides, GNIP1 promotes autophagy progression through mediating the K48-linked ubiquitination of 14-3-3ζ, which is a negative regulator of autophagy. In addition, highly expressed GNIP1 could promote NSCLC cells proliferation and migration. Clinically, a series of cohort analyses of NSCLC patients suggested that GNIP1 is a new prognostic indicator of NSCLC and can be used as an important anticancer target for NSCLC patients.

## Conclusions

Together, these findings demonstrated that GNIP1 is a novel regulatory factor of autophagy and promotes the proliferation and migration of NSCLC cells via autophagy. Our study for the first time revealed the role of GNIP1 as a tumor promoting factor in NSCLC, suggesting that GNIP1 could be a promising anti-tumor target in NSCLC.

## Supplementary Information

**Additional file 1:** Supplementary Figures 1-3.

**Additional file 2:** Supplementary Figure legends.

**Additional file 3:** Raw western blot data.

## Data Availability

All data generated or analysed during this study are included in this published article.

## Abbreviations

GNIP1: Glycogen interacting protein 1
NSCLC: Non-small cell lung cancer
TRIM: Tripartite motif
SQSTM1: Sequestosome 1
ATGs: Autophagy related genes
hVps34: Human vacuolar protein sorting 34
CHX: Cycloheximide
CQ: Chloroquine

## References

[1] Feng Y, He D, Yao Z, Klionsky DJ (2014). The machinery of macroautophagy. Cell Res.

[2] Parzych KR, Klionsky DJ (2014). An overview of autophagy: morphology, mechanism, and regulation. Antioxid Redox Signal.

[3] Choi AM, Ryter SW, Levine B (2013). Autophagy in human health and disease. N Engl J Med.

[4] Mizushima N (2007). Autophagy: process and function. Genes Dev.

[5] Aita VM, Liang XH, Murty VV, Pincus DL, Yu W, Cayanis E (1999). Cloning and genomic organization of beclin 1, a candidate tumor suppressor gene on chromosome 17q21. Genomics.

[6] Liang XH, Jackson S, Seaman M, Brown K, Kempkes B, Hibshoosh H (1999). Induction of autophagy and inhibition of tumorigenesis by beclin 1. Nature.

[7] Pozuelo-Rubio M (2011). 14-3-3zeta binds class III phosphatidylinositol-3-kinase and inhibits autophagy. Autophagy.

[8] Zhi X, Zhong Q (2015). Autophagy in cancer. F1000Prime Rep.

[9] Martinez J, Malireddi RK, Lu Q, Cunha LD, Pelletier S, Gingras S (2015). Molecular characterization of LC3-associated phagocytosis reveals distinct roles for Rubicon, NOX2 and autophagy proteins. Nat Cell Biol.

[10] Otomo C, Metlagel Z, Takaesu G, Otomo T (2013). Structure of the human ATG12~ATG5 conjugate required for LC3 lipidation in autophagy. Nat Struct Mol Biol.

[11] Pozuelo-Rubio M (2011). Regulation of autophagic activity by 14-3-3zeta proteins associated with class III phosphatidylinositol-3-kinase. Cell Death Differ.

[12] Zhai L, Dietrich A, Skurat AV, Roach PJ (2004). Structure-function analysis of GNIP, the glycogenin-interacting protein. Arch Biochem Biophys.

[13] Skurat AV, Dietrich AD, Zhai L, Roach PJ (2002). GNIP, a novel protein that binds and activates glycogenin, the self-glucosylating initiator of glycogen biosynthesis. J Biol Chem.

[14] D'Cruz AA, Babon JJ, Norton RS, Nicola NA, Nicholson SE (2013). Structure and function of the SPRY/B30.2 domain proteins involved in innate immunity. Protein Sci.

[15] Henry J, Mather IH, McDermott MF, Pontarotti P (1998). B30.2-like domain proteins: update and new insights into a rapidly expanding family of proteins. Mol Biol Evol.

[16] Henry J, Ribouchon MT, Offer C, Pontarotti P (1997). B30.2-like domain proteins: a growing family. Biochem Biophys Res Commun.

[17] Chakraborty A, Diefenbacher ME, Mylona A, Kassel O, Behrens A (2015). The E3 ubiquitin ligase Trim7 mediates c-Jun/AP-1 activation by Ras signalling. Nat Commun.

[18] Montori-Grau M, Pedreira-Casahuga R, Boyer-Diaz Z, Lassot I, Garcia-Martinez C, Orozco A (2018). GNIP1 E3 ubiquitin ligase is a novel player in regulating glycogen metabolism in skeletal muscle. Metabolism.

[19] Hu X, Tang Z, Ma S, Yu Y, Chen X, Zang G (2019). Tripartite motif-containing protein 7 regulates hepatocellular carcinoma cell proliferation via the DUSP6/p38 pathway. Biochem Biophys Res Commun.

[20] Jin J, Lu Z, Wang X, Liu Y, Han T, Wang Y (2020). E3 ubiquitin ligase TRIM7 negatively regulates NF-kappa B signaling pathway by degrading p65 in lung cancer. Cell Signal.

[21] Chauhan S, Kumar S, Jain A, Ponpuak M, Mudd MH, Kimura T (2016). TRIMs and galectins globally cooperate and TRIM16 and galectin-3 co-direct autophagy in endomembrane damage homeostasis. Dev Cell.

[22] Kimura T, Jain A, Choi SW, Mandell MA, Schroder K, Johansen T (2015). TRIM-mediated precision autophagy targets cytoplasmic regulators of innate immunity. J Cell Biol.

[23] Kimura T, Jia J, Kumar S, Choi SW, Gu Y, Mudd M (2017). Dedicated SNAREs and specialized TRIM cargo receptors mediate secretory autophagy. EMBO J.

[24] Liu T, Tang Q, Liu K, Xie W, Liu X, Wang H (2016). TRIM11 suppresses AIM2 inflammasome by degrading AIM2 via p62-dependent selective autophagy. Cell Rep.

[25] Mandell MA, Jain A, Arko-Mensah J, Chauhan S, Kimura T, Dinkins C (2014). TRIM proteins regulate autophagy and can target autophagic substrates by direct recognition. Dev Cell.

[26] Mandell MA, Jain A, Kumar S, Castleman MJ, Anwar T, Eskelinen EL (2016). TRIM17 contributes to autophagy of midbodies while actively sparing other targets from degradation. J Cell Sci.

[27] Pineda CT, Potts PR (2015). Oncogenic MAGEA-TRIM28 ubiquitin ligase downregulates autophagy by ubiquitinating and degrading AMPK in cancer. Autophagy.

[28] Ra EA, Lee TA, Won Kim S, Park A, Choi HJ, Jang I (2016). TRIM31 promotes Atg5/Atg7-independent autophagy in intestinal cells. Nat Commun.

[29] Sparrer KMJ, Gableske S, Zurenski MA, Parker ZM, Full F, Baumgart GJ (2017). TRIM23 mediates virus-induced autophagy via activation of TBK1. Nat Microbiol.

[30] Tomar D, Prajapati P, Sripada L, Singh K, Singh R, Singh AK (2013). TRIM13 regulates caspase-8 ubiquitination, translocation to autophagosomes and activation during ER stress induced cell death. Biochim Biophys Acta.

[31] Tomar D, Singh R, Singh AK, Pandya CD, Singh R (2012). TRIM13 regulates ER stress induced autophagy and clonogenic ability of the cells. Biochim Biophys Acta.

[32] Hatakeyama S (2017). TRIM family proteins: roles in autophagy, immunity, and carcinogenesis. Trends Biochem Sci.

[33] Klionsky DJ, Abdel-Aziz AK, Abdelfatah S, Abdellatif M, Abdoli A, Abel S (2021). Guidelines for the use and interpretation of assays for monitoring autophagy (4th edition)(1). Autophagy.

[34] Aitken A (2011). Post-translational modification of 14-3-3 isoforms and regulation of cellular function. Semin Cell Dev Biol.

[35] Matta A, Siu KW, Ralhan R (2012). 14-3-3 zeta as novel molecular target for cancer therapy. Expert Opin Ther Targets.

[36] Yang X, Lee WH, Sobott F, Papagrigoriou E, Robinson CV, Grossmann JG (2006). Structural basis for protein-protein interactions in the 14-3-3 protein family. Proc Natl Acad Sci U S A.

[37] Guo F, Jiao D, Sui GQ, Sun LN, Gao YJ, Fu QF (2018). Anticancer effect of YWHAZ silencing via inducing apoptosis and autophagy in gastric cancer cells. Neoplasma.

[38] Swatek KN, Komander D (2016). Ubiquitin modifications. Cell Res.

[39] Ciechanover A (2015). The unravelling of the ubiquitin system. Nat Rev Mol Cell Biol.

[40] Chun Y, Kim J (2018). Autophagy: an essential degradation program for cellular homeostasis and life. Cells.

[41] Corona Velazquez AF, Jackson WT (2018). So many roads: the multifaceted regulation of autophagy induction. Mol Cell Biol.

[42] Li Y, Chen Y (2019). AMPK and Autophagy. Adv Exp Med Biol.

[43] Proikas-Cezanne T, Takacs Z, Donnes P, Kohlbacher O (2015). WIPI proteins: essential PtdIns3P effectors at the nascent autophagosome. J Cell Sci.

[44] Mizushima N, Yoshimori T, Ohsumi Y (2011). The role of Atg proteins in autophagosome formation. Annu Rev Cell Dev Biol.

[45] Hatakeyama S (2011). TRIM proteins and cancer. Nat Rev Cancer.

[46] Meroni G, Diez-Roux G (2005). TRIM/RBCC, a novel class of ‘single protein RING finger’ E3 ubiquitin ligases. BioEssays.

[47] Ozato K, Shin DM, Chang TH, Morse HC (2008). TRIM family proteins and their emerging roles in innate immunity. Nat Rev Immunol.

[48] Han T, Guo M, Gan M, Yu B, Tian X, Wang JB (2018). TRIM59 regulates autophagy through modulating both the transcription and the ubiquitination of BECN1. Autophagy.

[49] Morrison DK (2009). The 14-3-3 proteins: integrators of diverse signaling cues that impact cell fate and cancer development. Trends Cell Biol.

[50] Kilani RT, Medina A, Aitken A, Jalili RB, Carr M, Ghahary A (2008). Identification of different isoforms of 14-3-3 protein family in human dermal and epidermal layers. Mol Cell Biochem.

