# Supplementary material for: GNIP1 functions both as a scaffold protein and an E3 ubiquitin ligase to regulate autophagy in lung cancer
Source: Cell Commun Signal. 2022 Aug 30;20:133. doi: 10.1186/s12964-022-00936-x (PMC9426035; doi:10.1186/s12964-022-00936-x)

Supplementary Figures 1-3

Figure S1. Overexpression efficiency of GNIP1

A

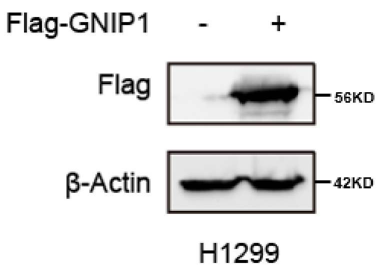

B

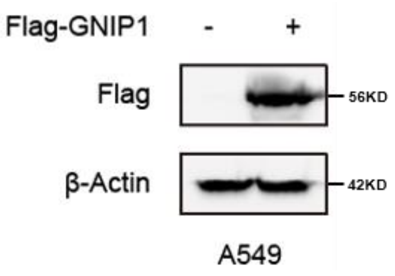

Figure S2. Overexpression efficiency of GNIP1 and GNIP1-W57A

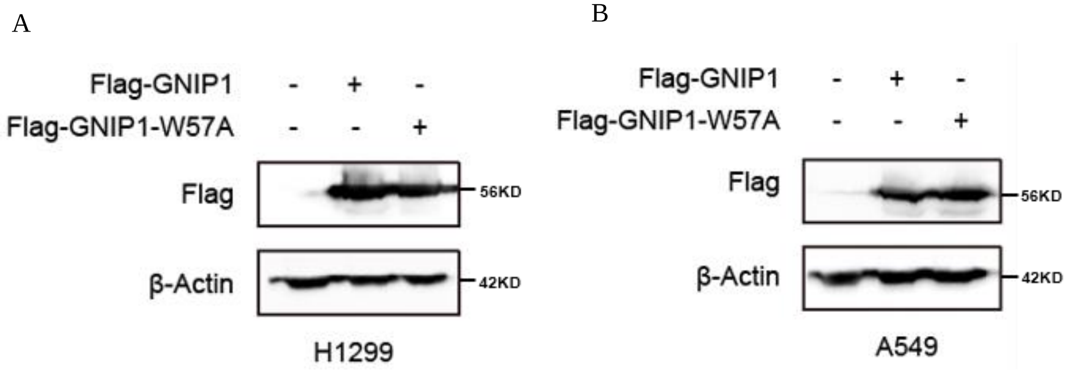

Figure S3. The efficiency of CQ inhibition and BECN1 siRNA knockdown

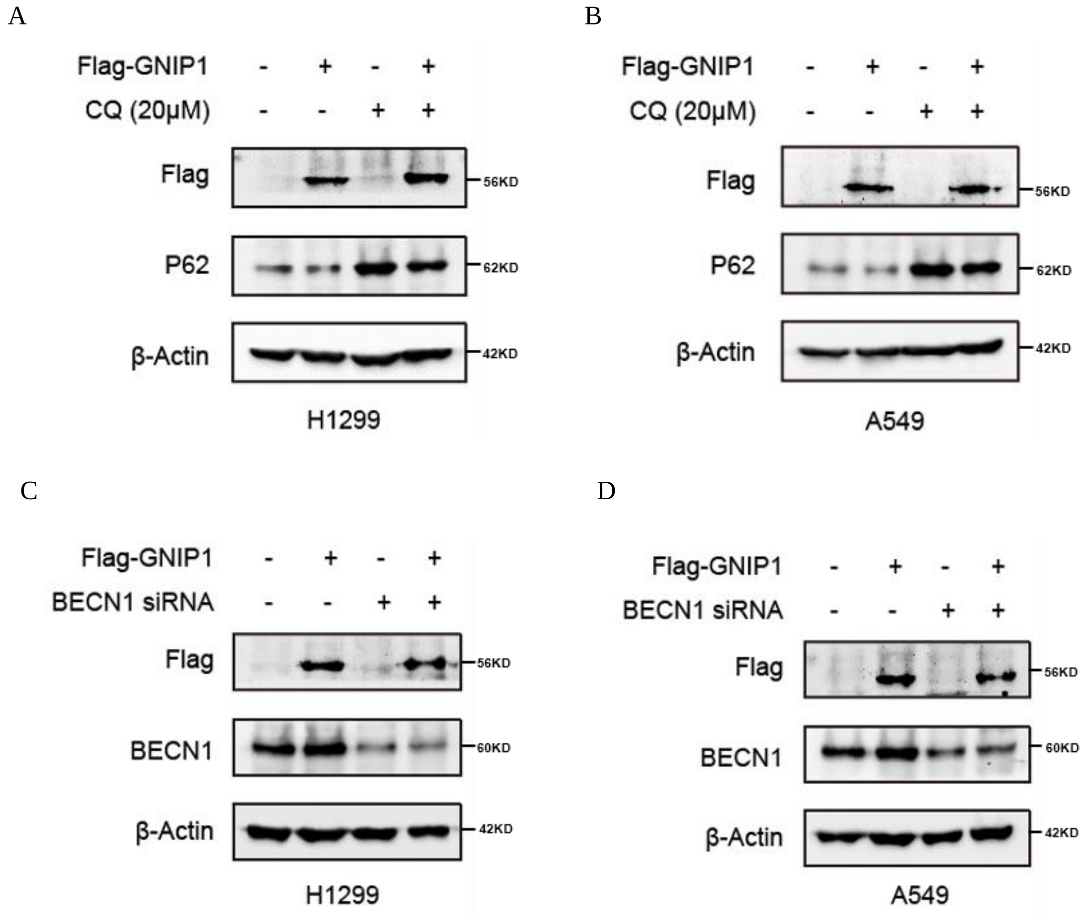

Supplement: Supplementary file 2 — Additional file 1: Supplementary Figures 1-3. [file 12964_2022_936_MOESM2_ESM.pdf]
