# Supplementary material for: GNIP1 functions both as a scaffold protein and an E3 ubiquitin ligase to regulate autophagy in lung cancer
Source: Cell Commun Signal. 2022 Aug 30;20:133. doi: 10.1186/s12964-022-00936-x (PMC9426035; doi:10.1186/s12964-022-00936-x)
Supplement: Supplementary file 3 — Additional file 2: Supplementary Figure legends. [file 12964_2022_936_MOESM3_ESM.pdf]

Supplementary Figure legends

Figure S1. Overexpression effect of GNIP1

H1299 cells (A) and A549 cells (B) were transfected with control empty vector or GNIP1 plasmids. The transfection efficiency of GNIP1 was detected by western blot.

Figure S2. Overexpression effect of GNIP1 and GNIP1-W57A

H1299 cells (A) and A549 cells (B) were transfected with GNIP1 or GNIP1-W57A plasmids. The transfection efficiency of GNIP1 or GNIP1-W57A was detected by western blot.

Figure S3. The efficiency of CQ inhibition and BECN1 siRNA interference

H1299 cells (A) and A549 cells (B) were transfected with empty vector or GNIP1 plasmids, the cells were treated with or without 20 μM chloroquine (CQ) for 12h. The efficiency of CQ inhibition and GNIP1 overexpression in different cell lines were detected by Western blot. H1299 cells (C) and A549 cells (D) were transfected with empty vector and GNIP1 plasmid or siRNA ctrl and siRNA BECN1. The efficiency of BECN1 interference and GNIP1 overexpression in different cell lines were detected by Western blot.
