# Supplementary material for: GNIP1 functions both as a scaffold protein and an E3 ubiquitin ligase to regulate autophagy in lung cancer
Source: Cell Commun Signal. 2022 Aug 30;20:133. doi: 10.1186/s12964-022-00936-x (PMC9426035; doi:10.1186/s12964-022-00936-x)
Supplement: Supplementary file 4 — Additional file 3: Raw western blot data. [file 12964_2022_936_MOESM4_ESM.pdf]

Raw western blot data

Fig 1F

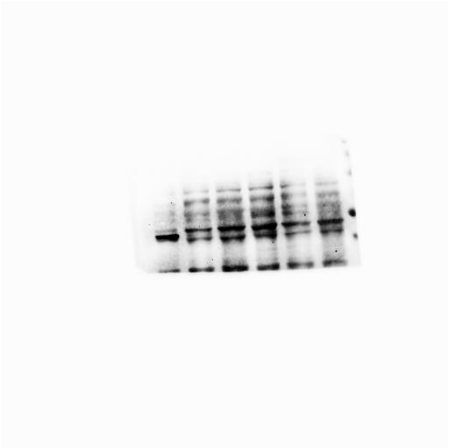

GNIP1

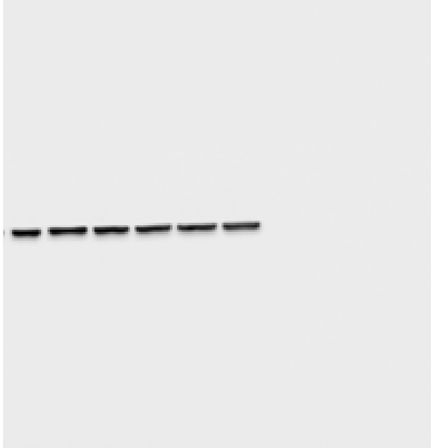

β-Actin

Fig 1G

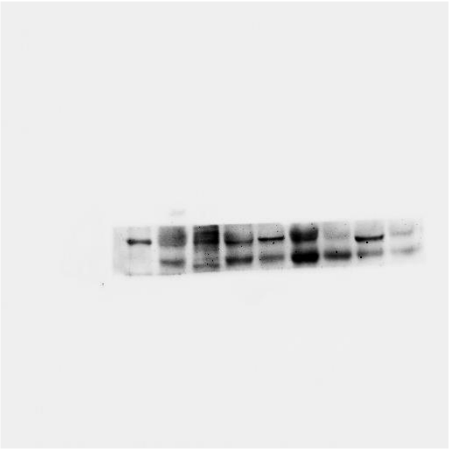

GNIP1

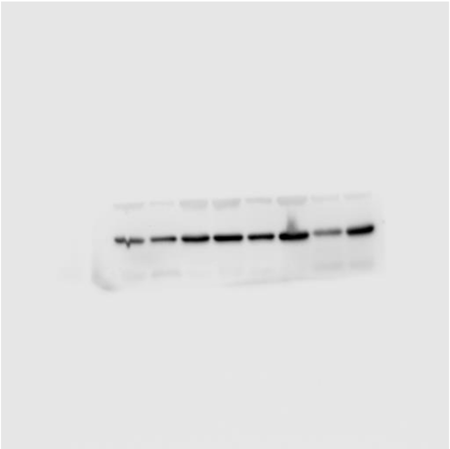

GAPDH

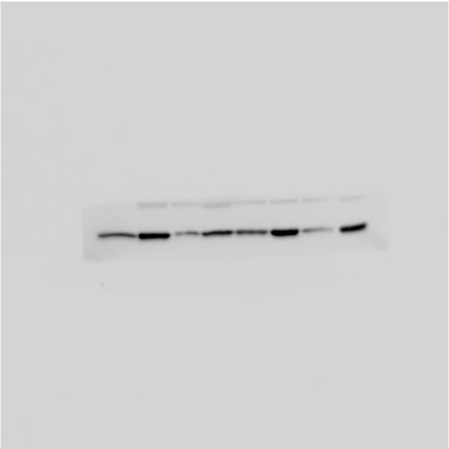

GNIP1

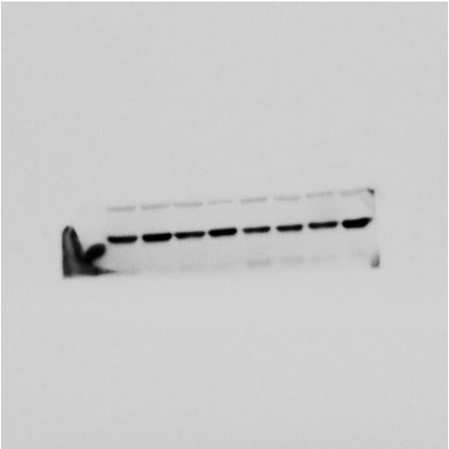

GAPDH

Fig 3A

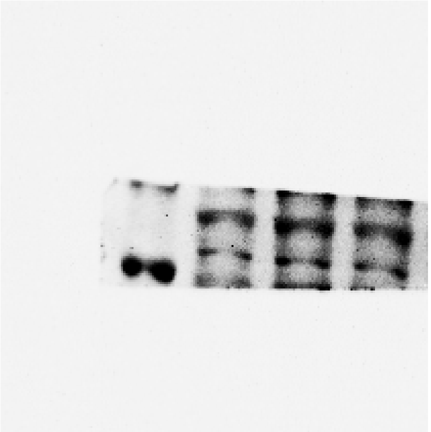

GNIP1

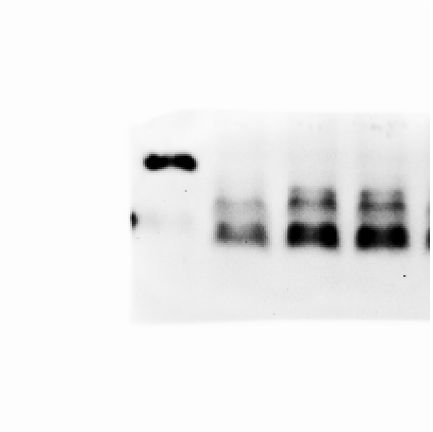

LC3B

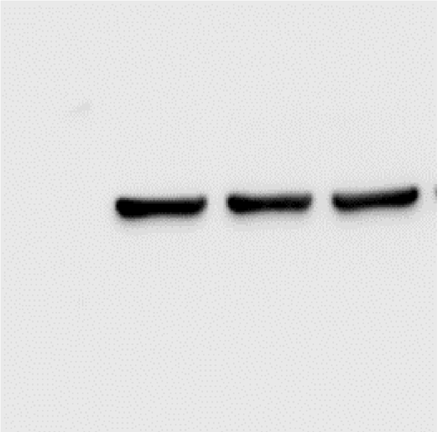

β-Actin

Fig 3B

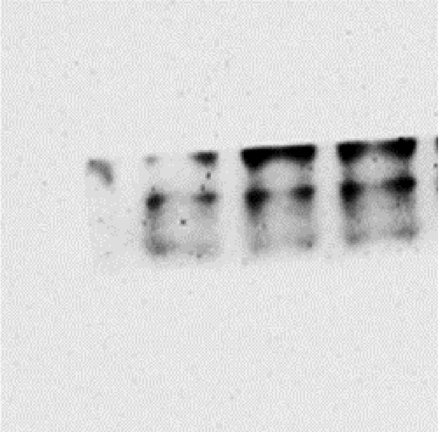

GNIP1

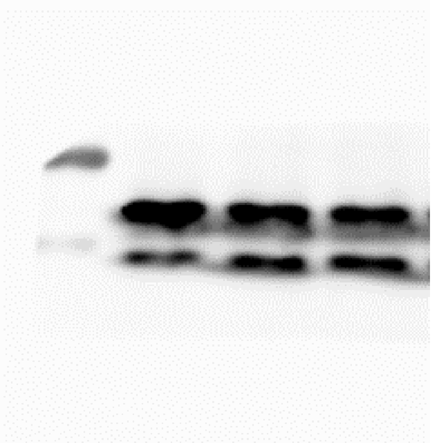

LC3B

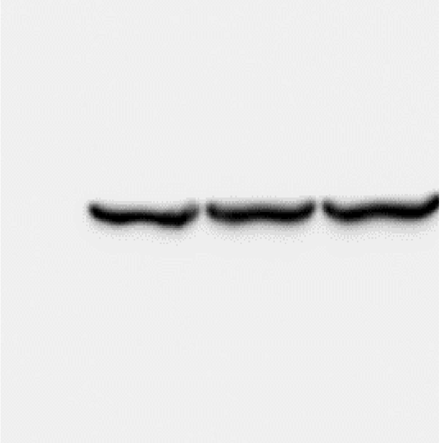

β-Actin

Fig 3E

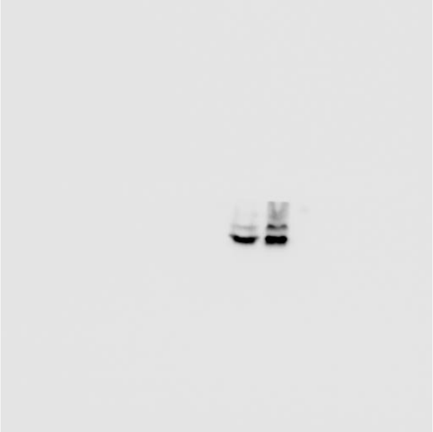

LC3B

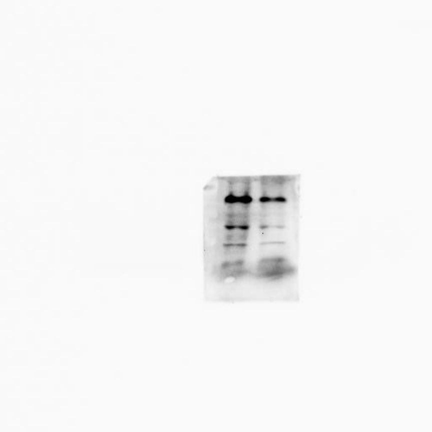

P62

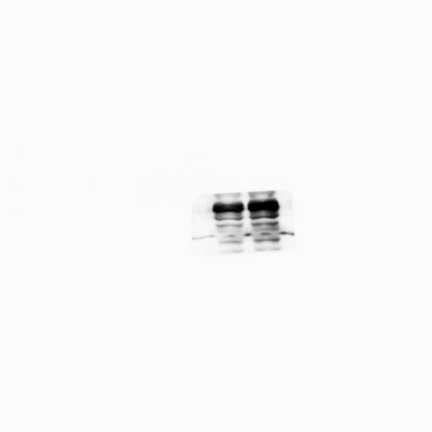

BECN1

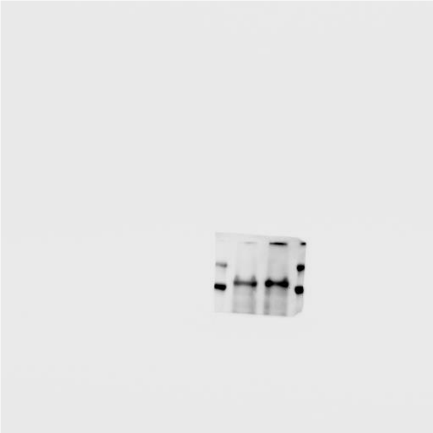

ULK1

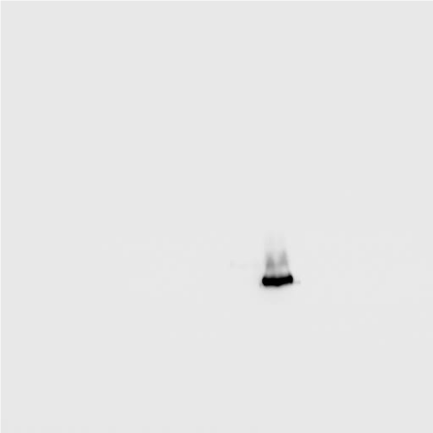

Flag

β-Actin

Fig 3F

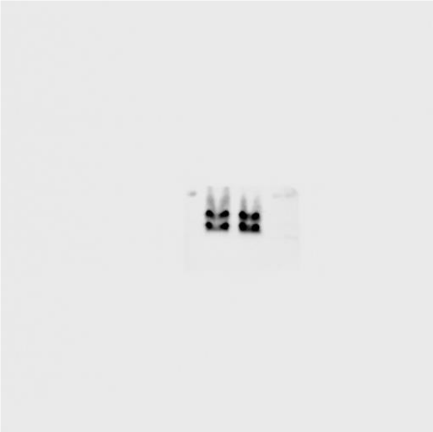

LC3B

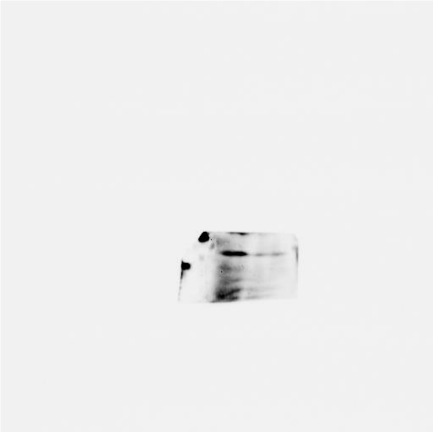

P62

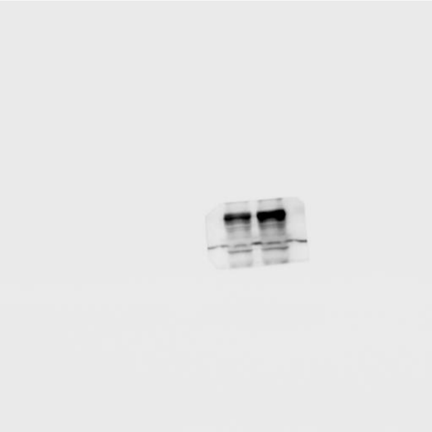

BECN1

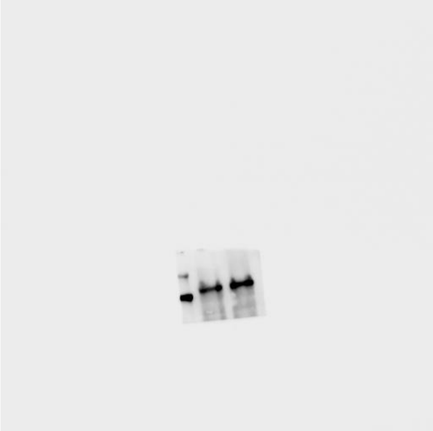

ULK1

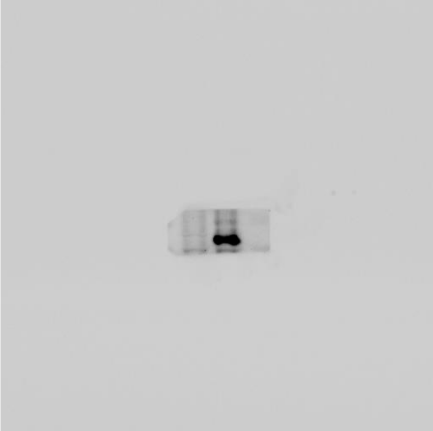

Flag

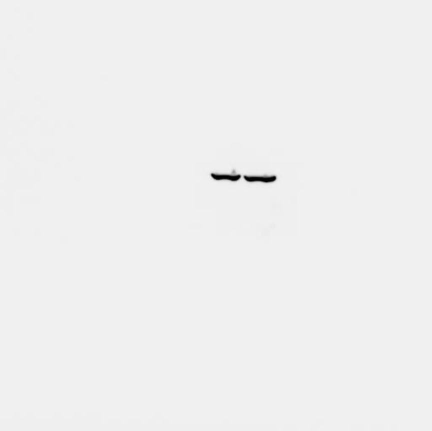

β-Actin

Fig 3G

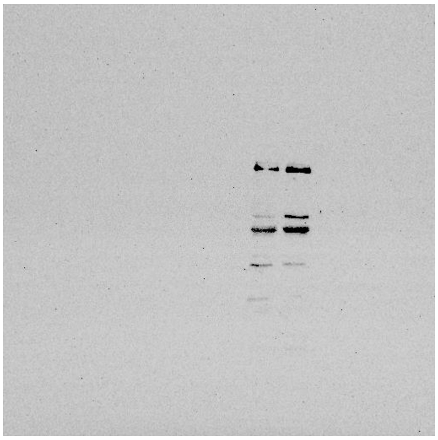

Flag

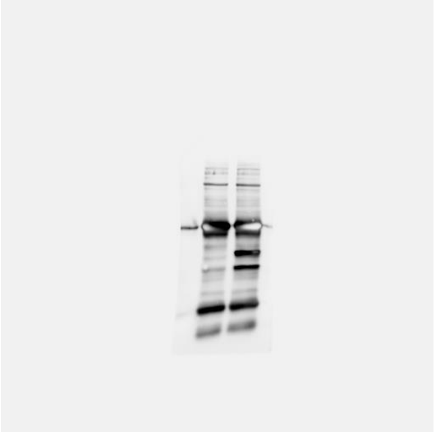

GFP

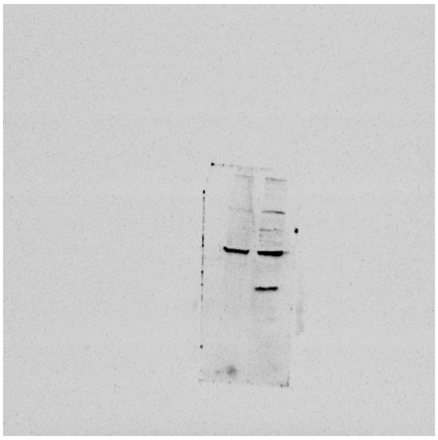

Flag

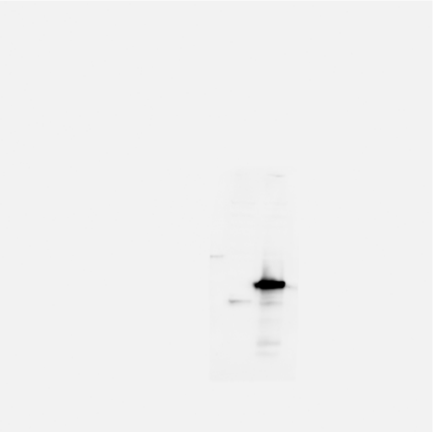

GFP

Fig 3H

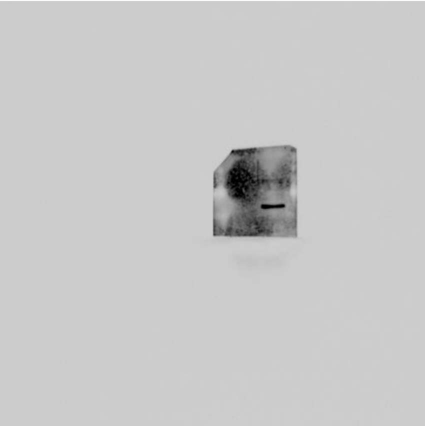

GFP

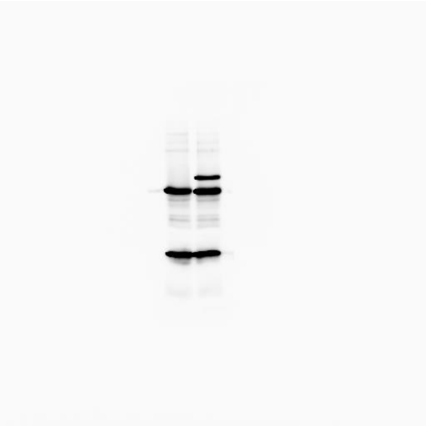

Flag

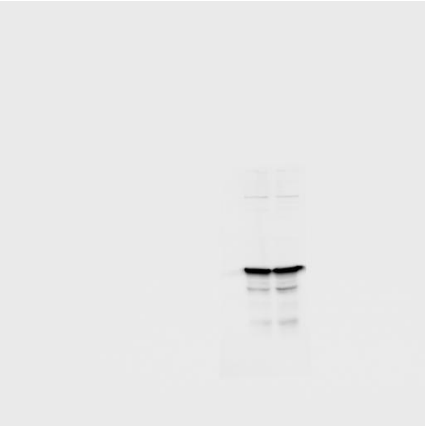

GFP

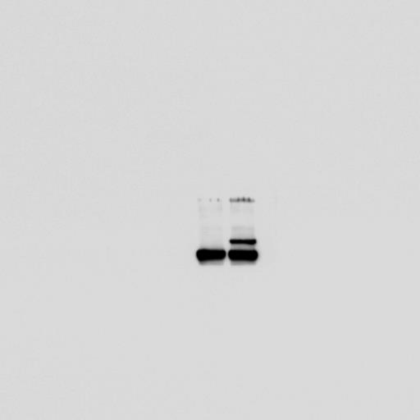

Flag

Fig 3I

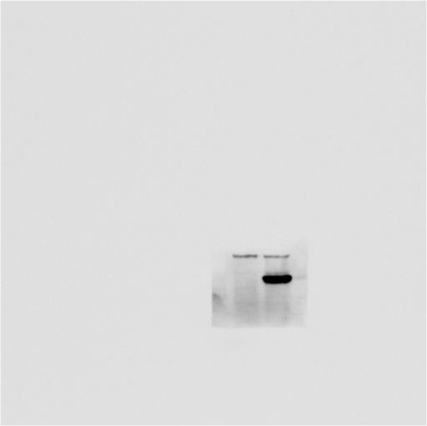

HA

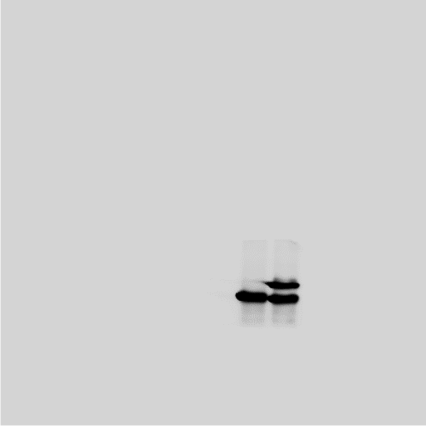

Flag

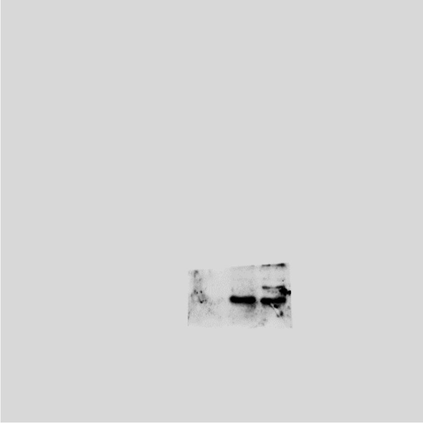

HA

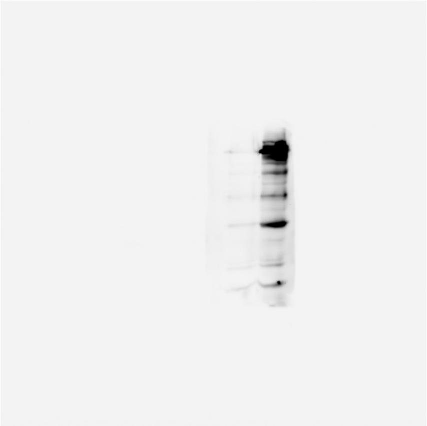

Flag

Fig 3K

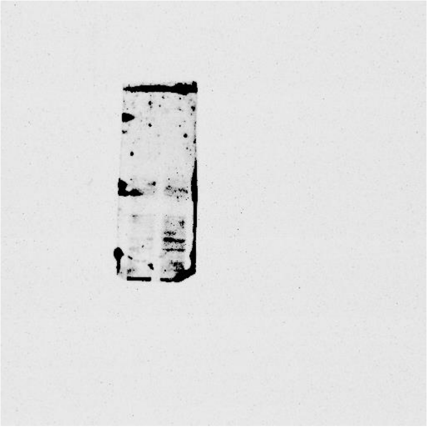

His

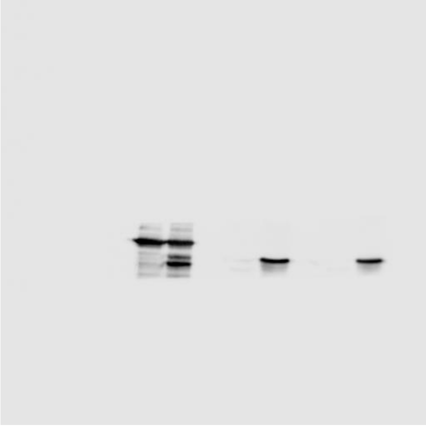

GFP

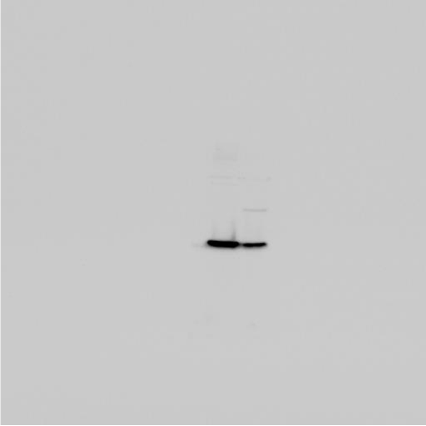

His

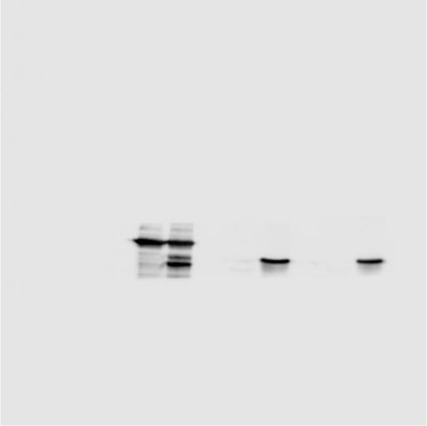

GFP

Fig 3L

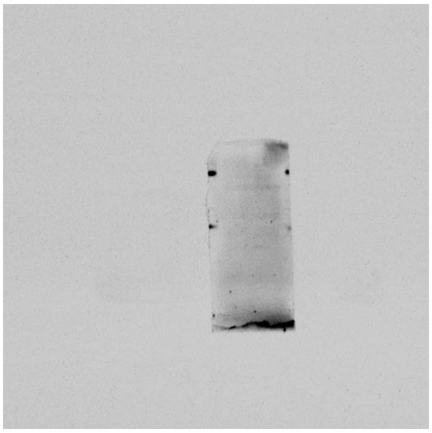

His

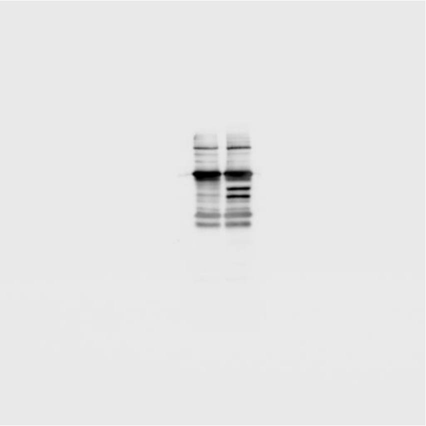

GFP

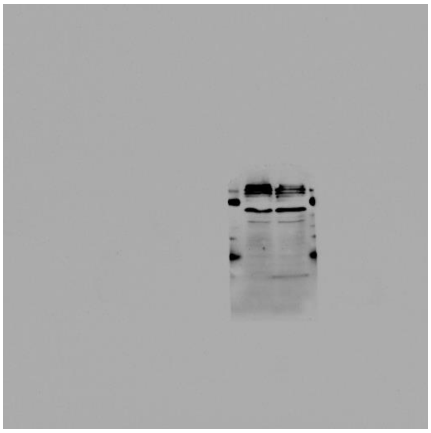

His

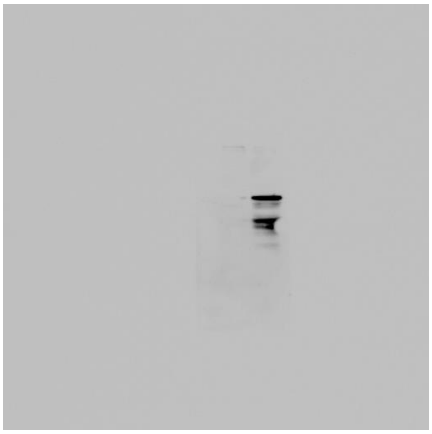

GFP

Fig 3M

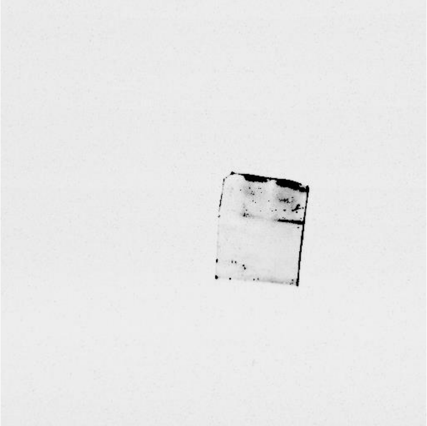

V5

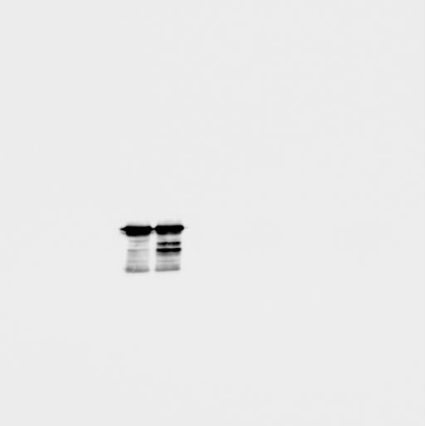

GFP

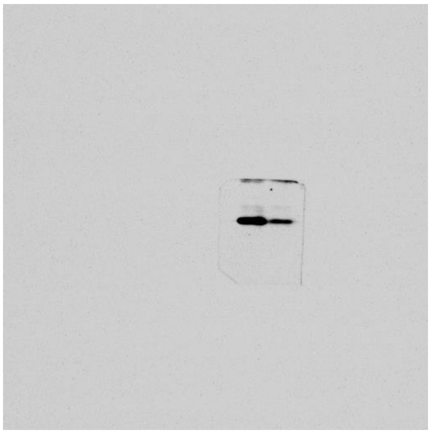

V5

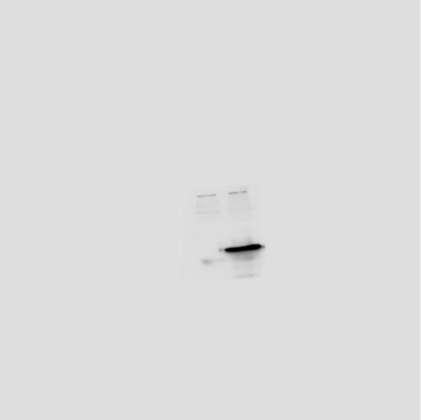

GFP

Fig 3N

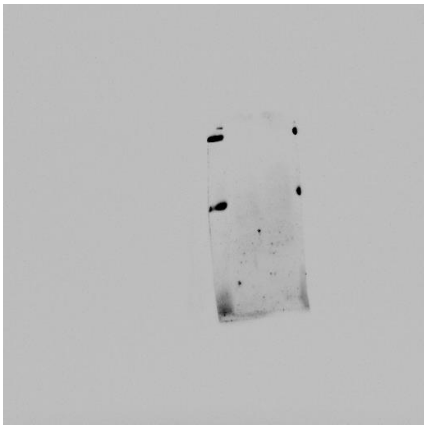

His

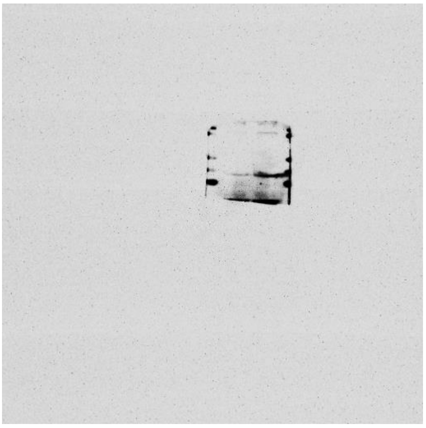

HA

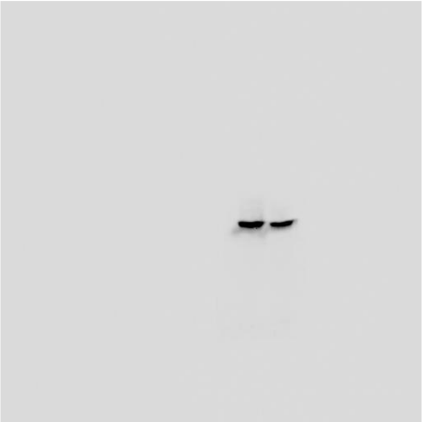

His

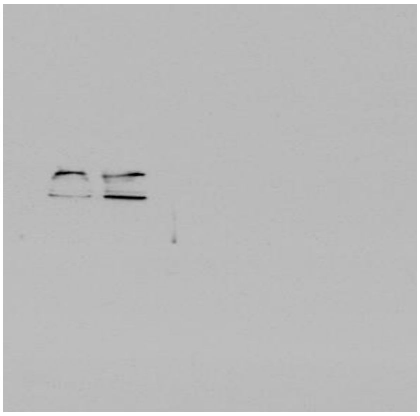

HA

Fig 3O

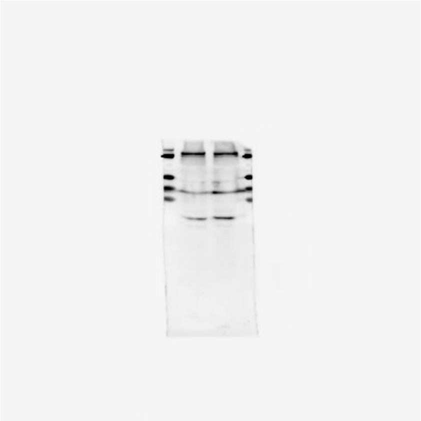

HA

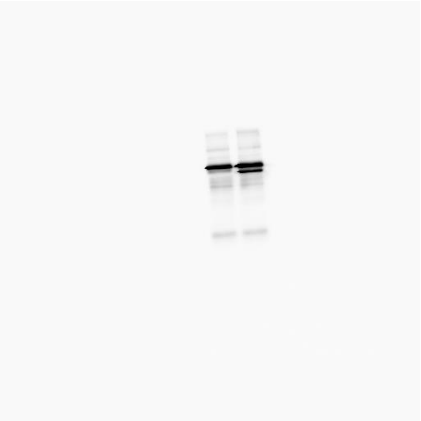

His

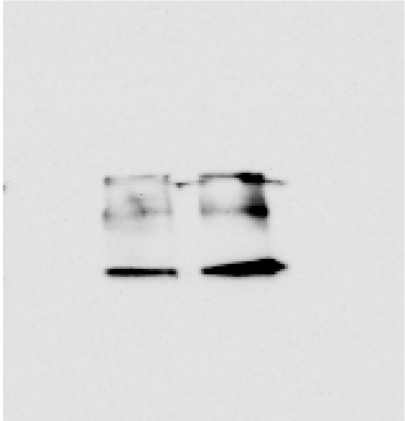

HA

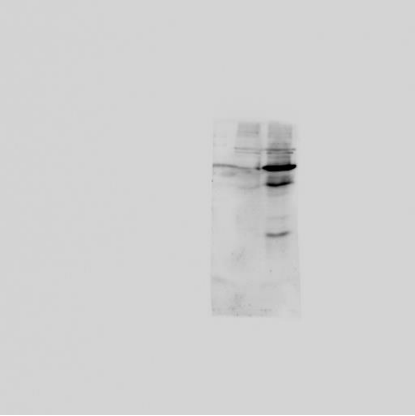

His

Fig 3P

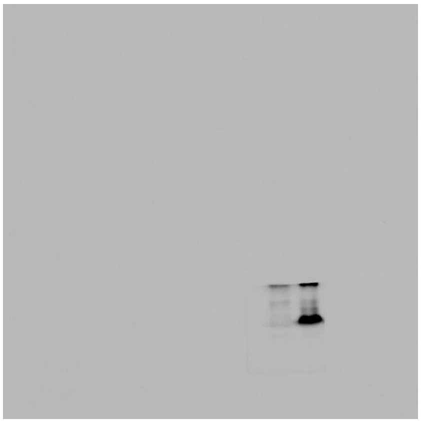

V5

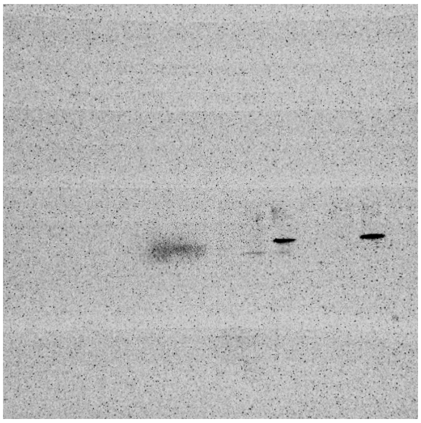

HA

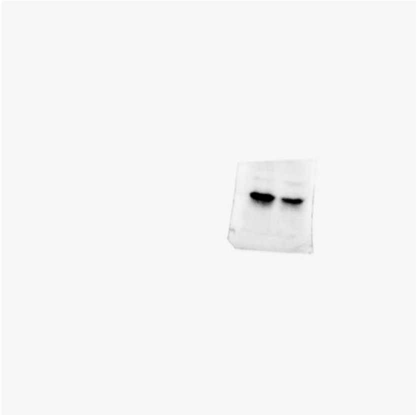

V5

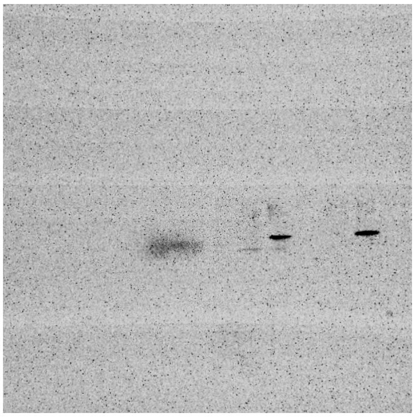

HA

Fig 4A

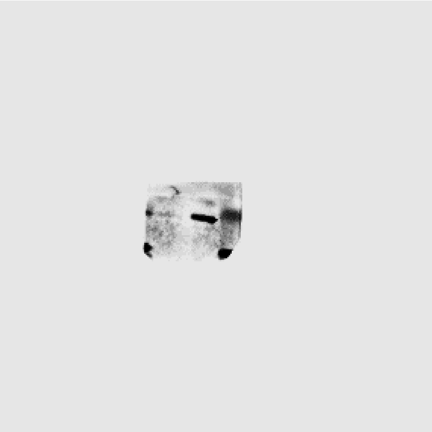

14-3-3ζ

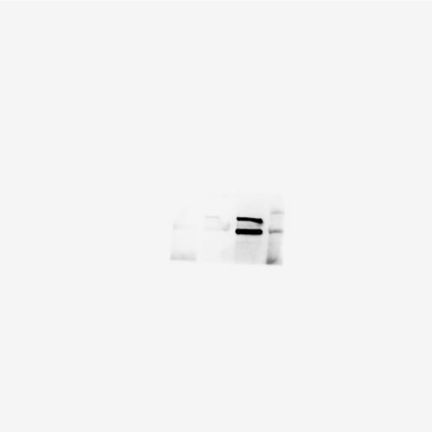

Flag

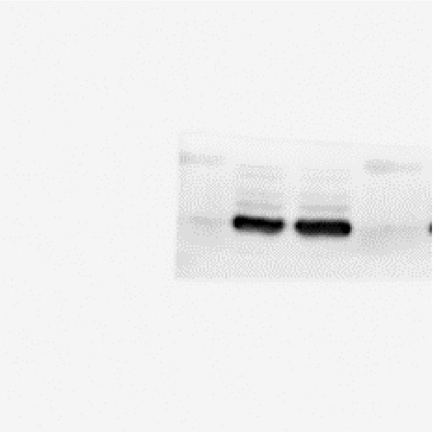

14-3-3ζ

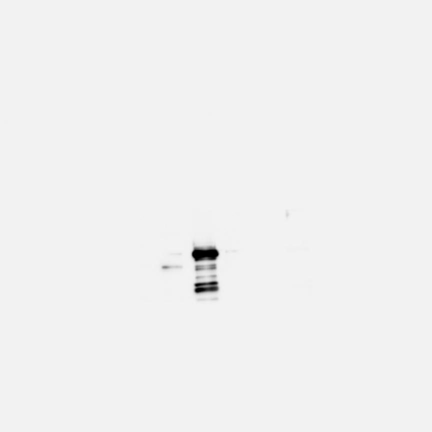

Flag

Fig 4B

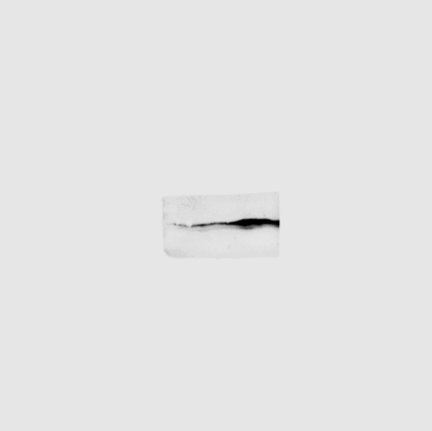

14-3-3ζ

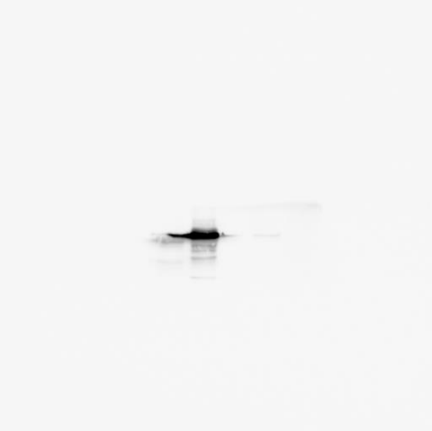

Flag

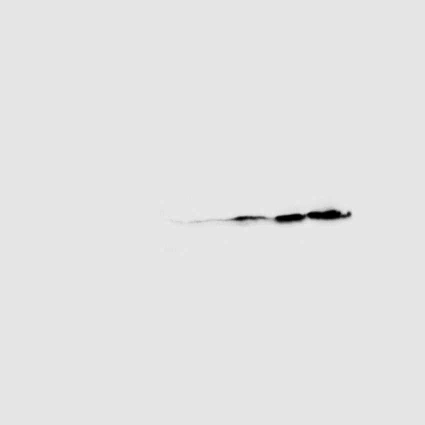

14-3-3ζ

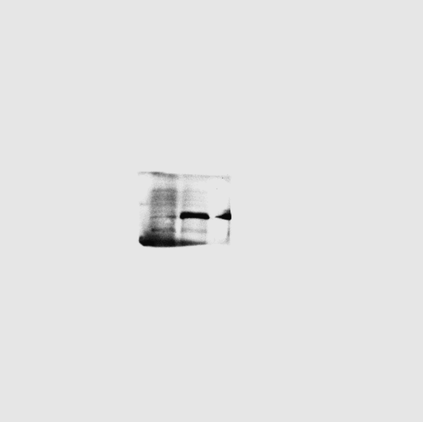

Flag

Fig 4C

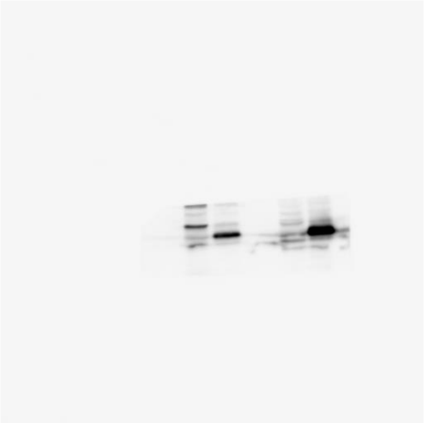

Flag

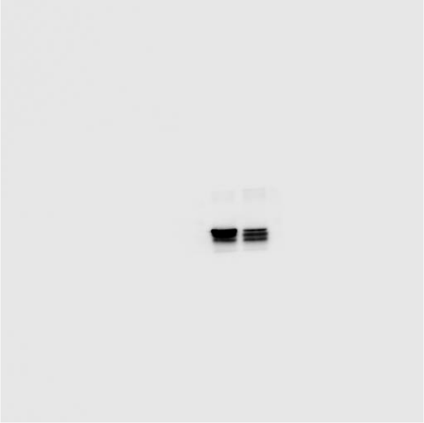

Myc

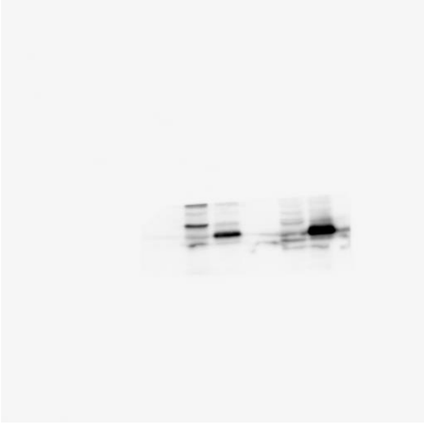

Flag

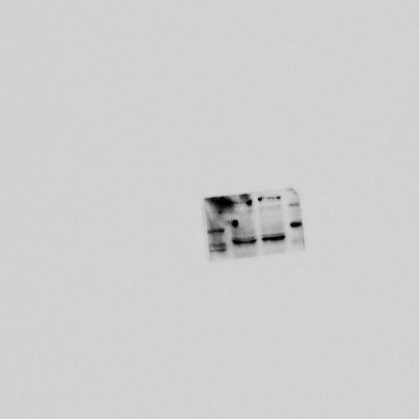

Myc

Fig 4D

14-3-3ζ

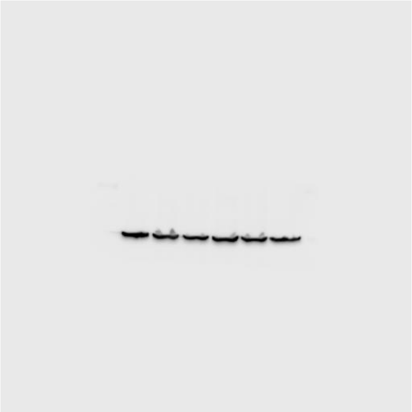

β-Actin

Fig 4E

14-3-3ζ

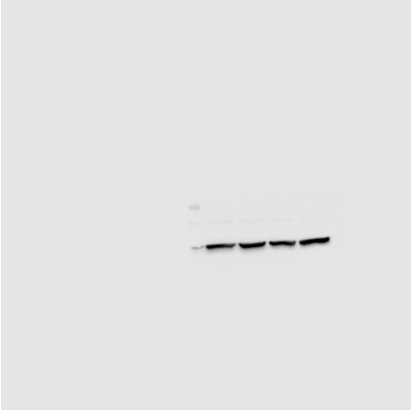

β-Actin

Fig 4F

14-3-3ζ

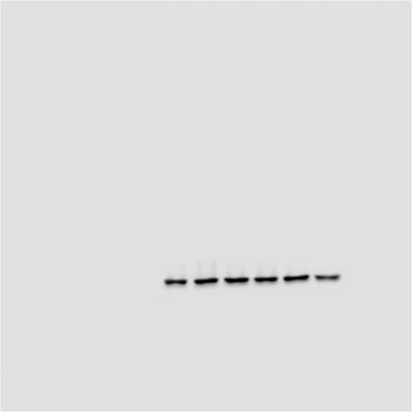

14-3-3ζ

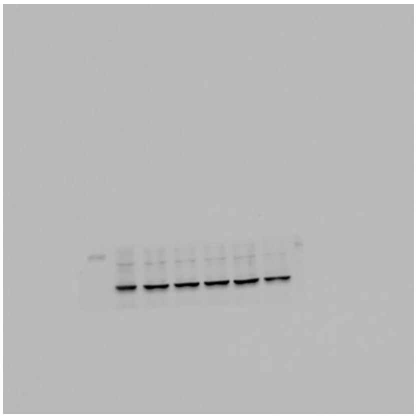

β-Actin

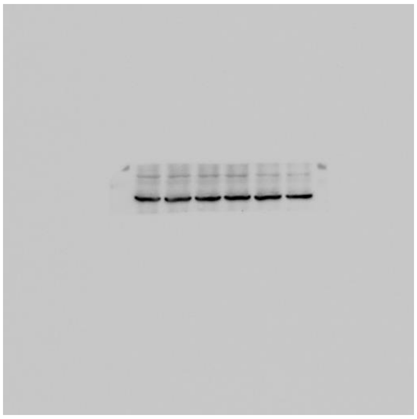

β-Actin

Fig 4G

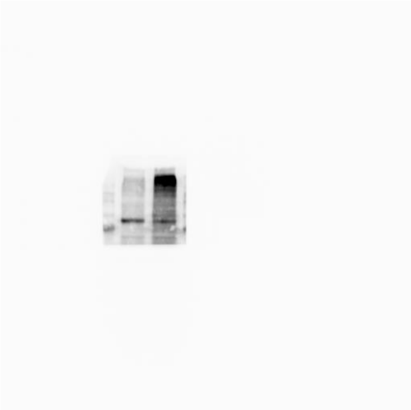

UB-14-3-3ζ

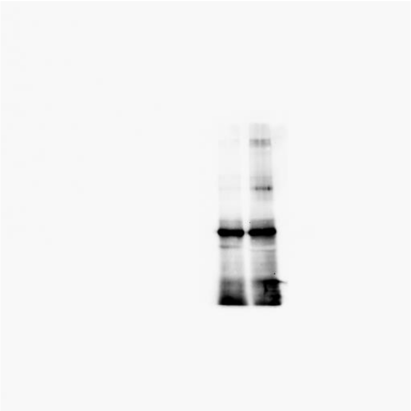

14-3-3ζ

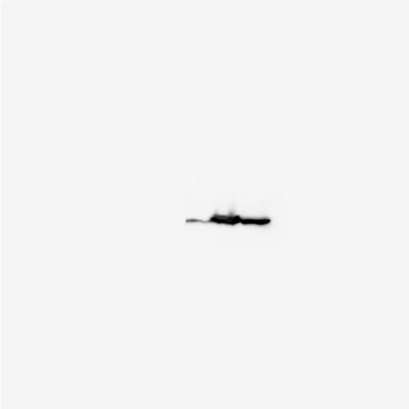

14-3-3ζ

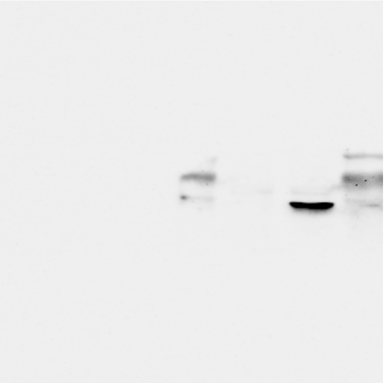

Flag-GNIP1

Flag- 14-3-3ζ

Fig 4H

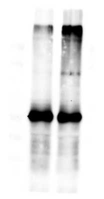

K48-14-3-3ζ

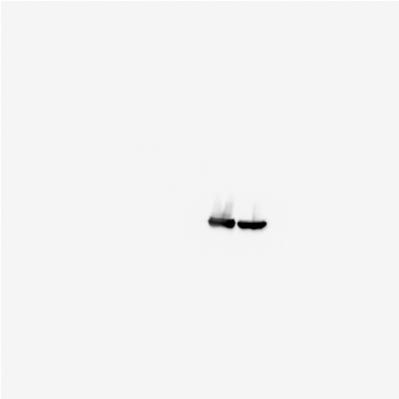

14-3-3ζ

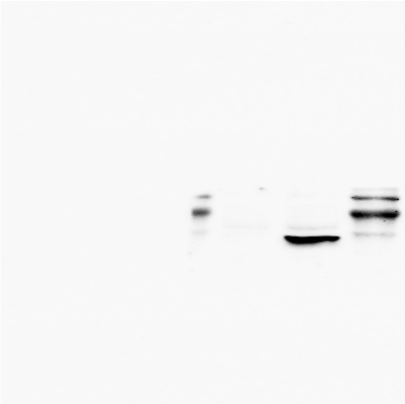

Flag-GNIP1

Flag- 14-3-3ζ

Fig 4I

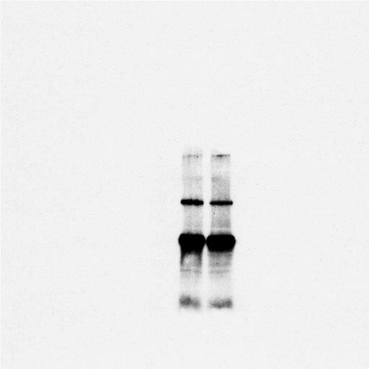

K63-Ub

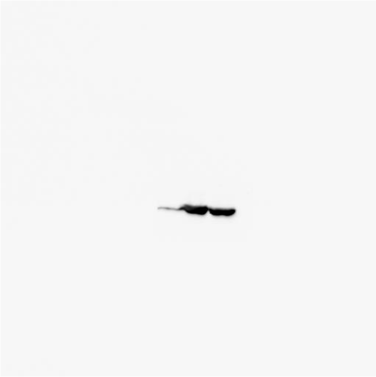

14-3-3ζ

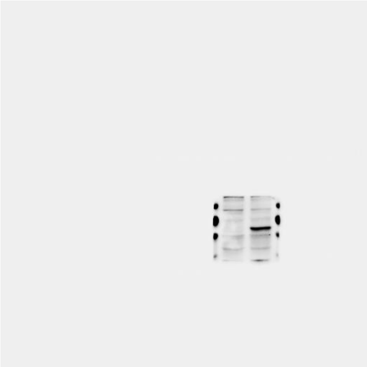

Flag-GNIP1

Flag- 14-3-3ζ

Fig 4J

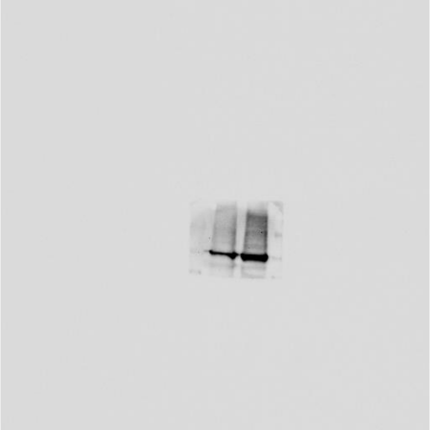

VPS34

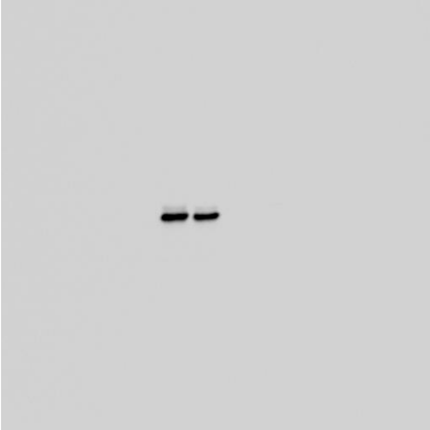

HA

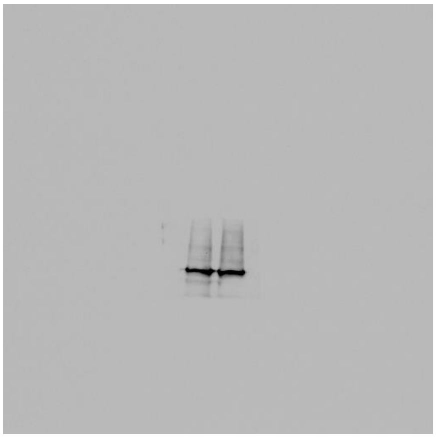

VPS34

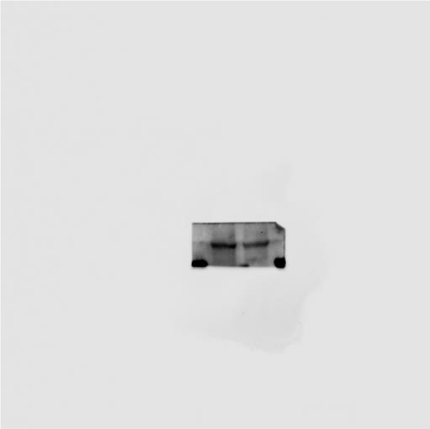

HA

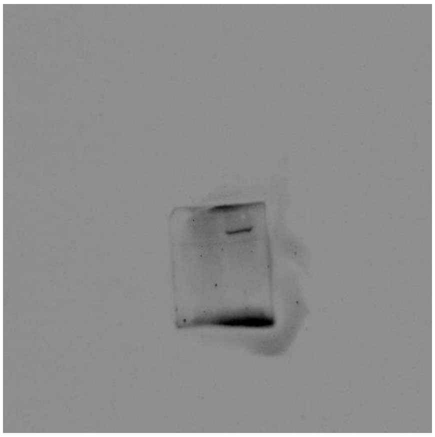

Flag

Fig 4K

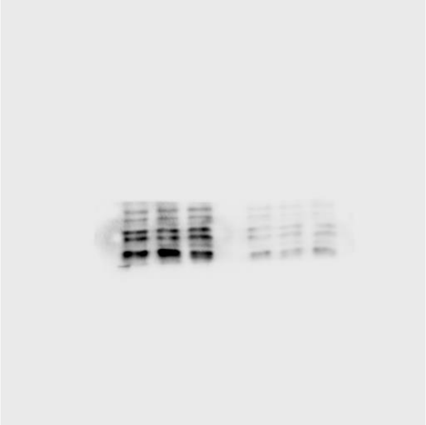

LC3B

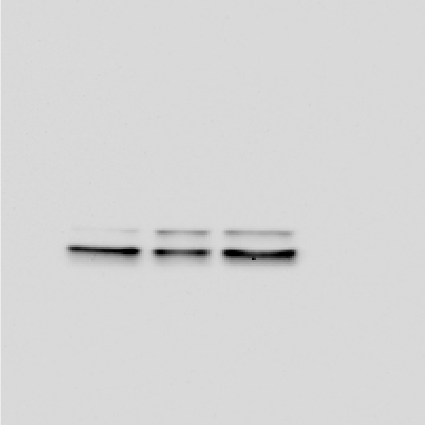

P62

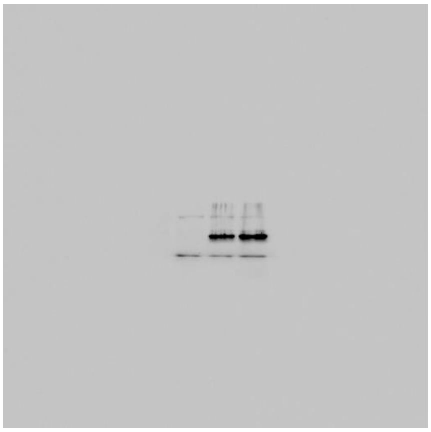

Flag-GNIP1

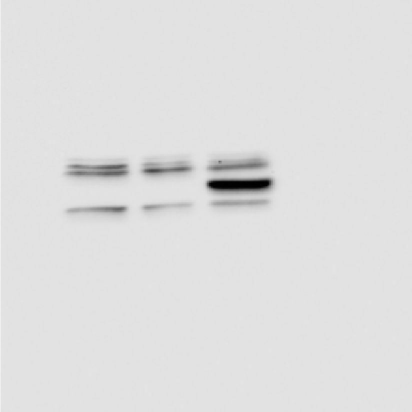

Flag- 14-3-3ζ

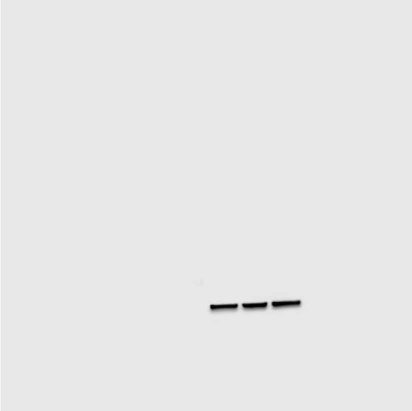

β-Actin

Fig 5A

14-3-3ζ

Flag

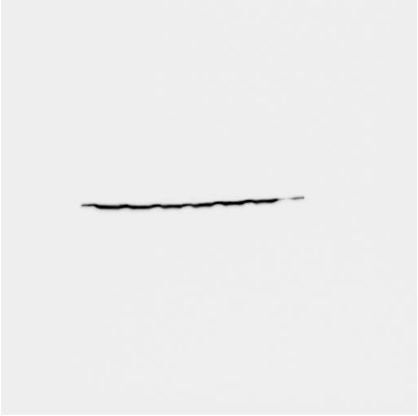
β-Actin

14-3-3ζ

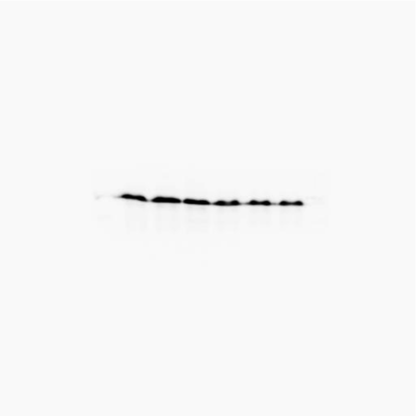
Flag

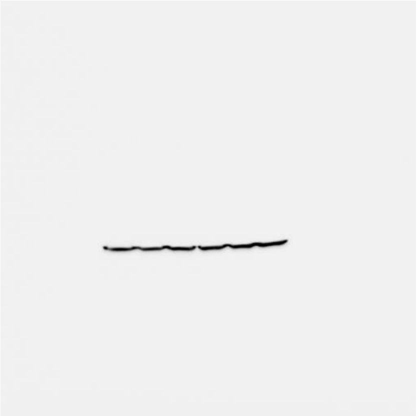
β-Actin

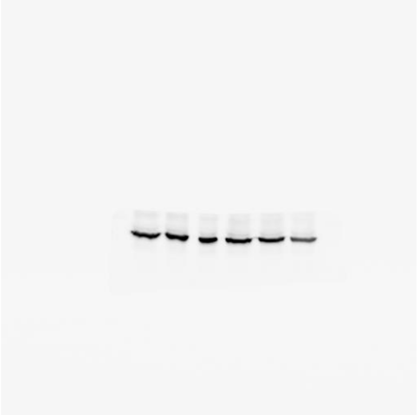
14-3-3ζ

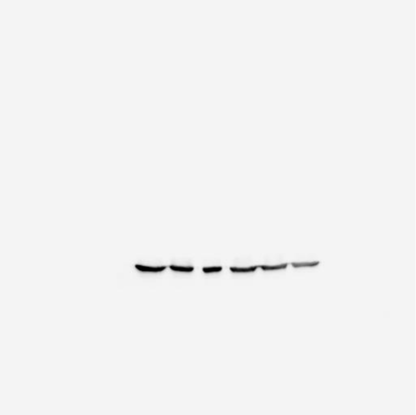
Flag

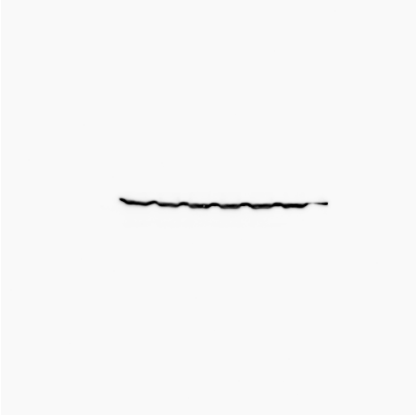
β-Actin

Fig 5B

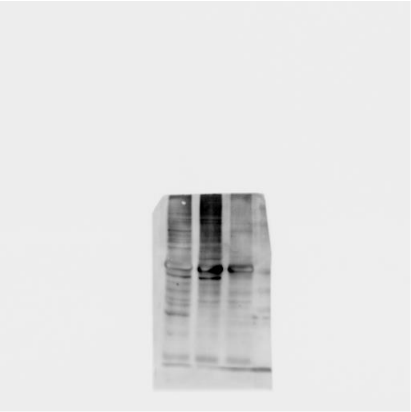

UB

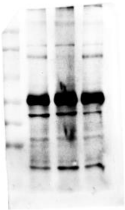

14-3-3ζ

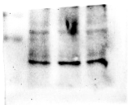

14-3-3ζ

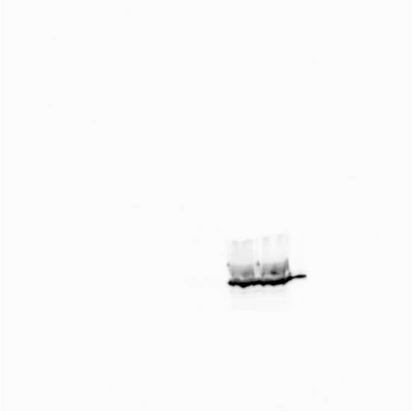

Flag-GNIP1

Flag- 14-3-3ζ

Fig 5C

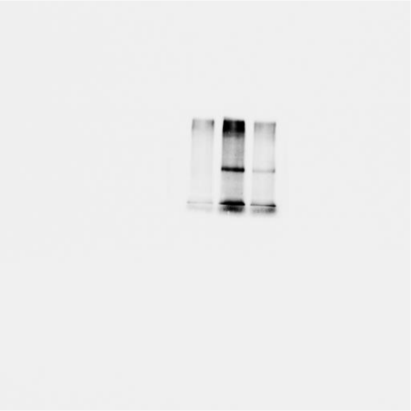

K48

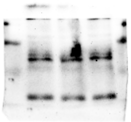

14-3-3ζ

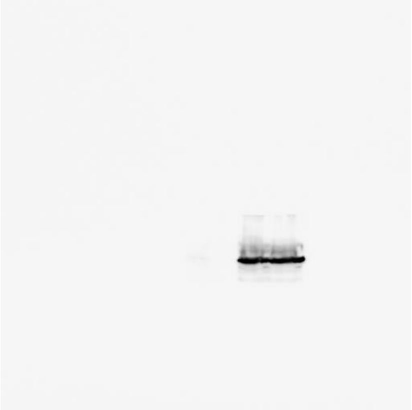

Flag-GNIP1

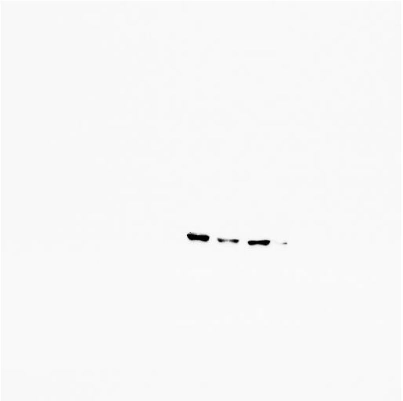

Flag- 14-3-3ζ

Fig 5D

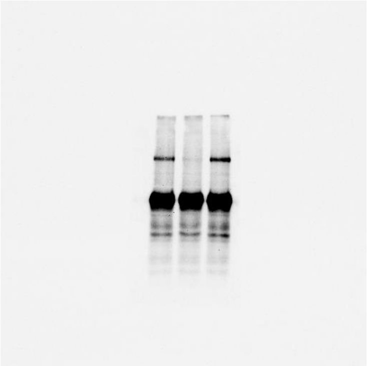

K63

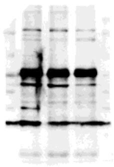

14-3-3ζ

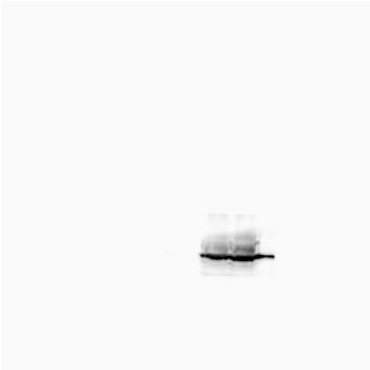

Flag-GNIP1

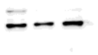

Flag- 14-3-3ζ

Fig 5E

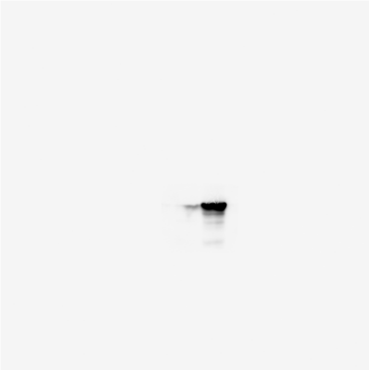

Flag

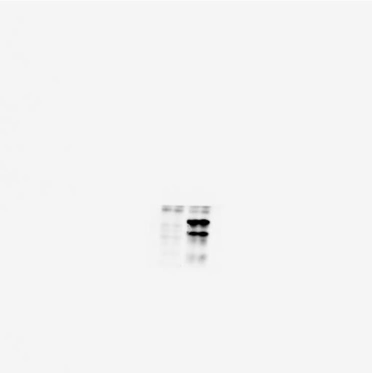

GFP

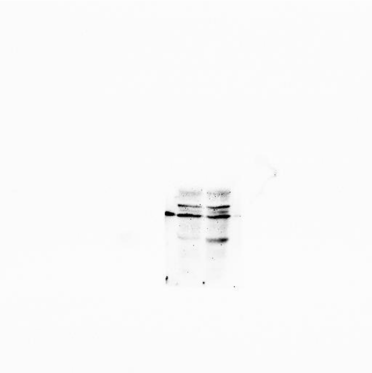

Flag

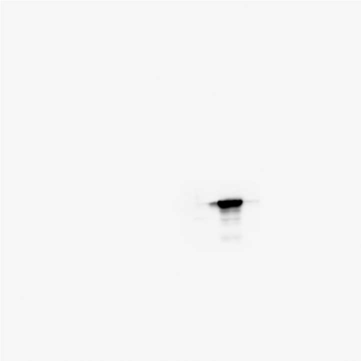

GFP

Fig 5F

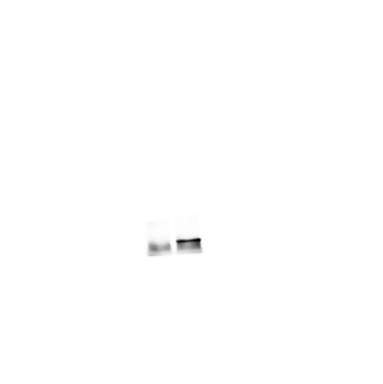

Flag

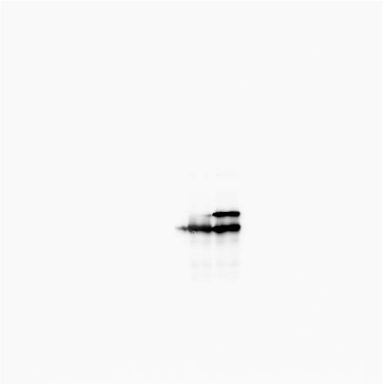

HA

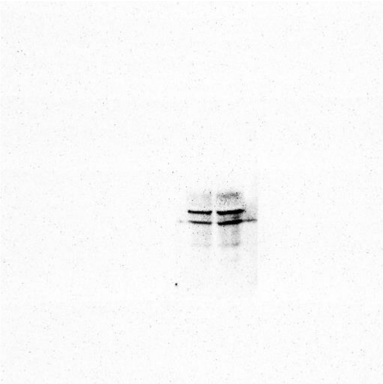

Flag

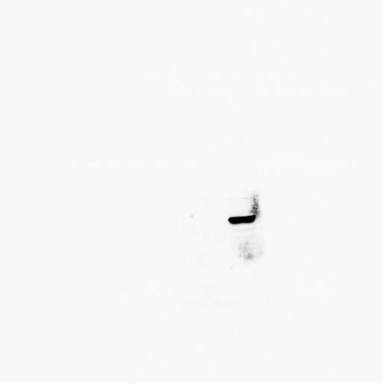

HA

Fig 7B

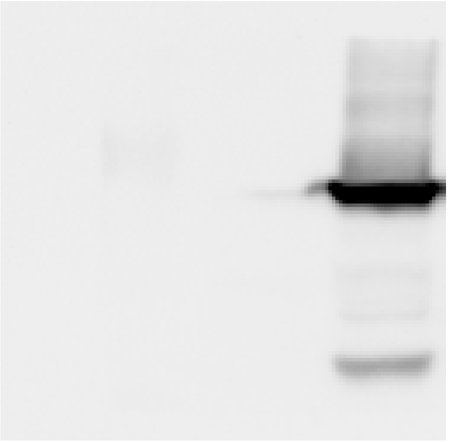

Flag

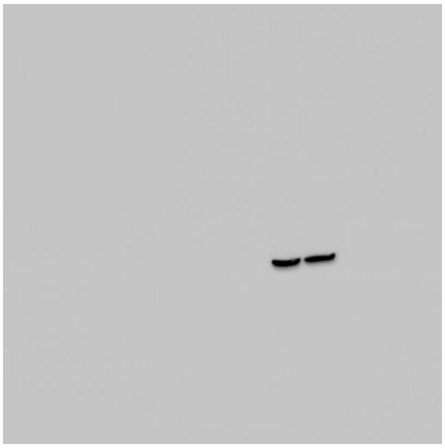

β-Actin

Fig S1A

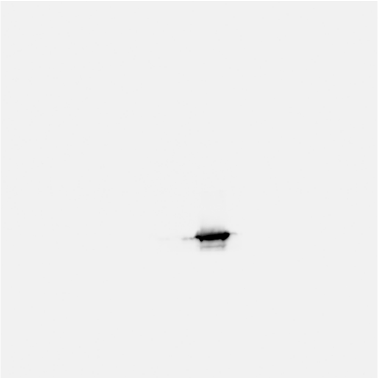

Flag

β-Actin

Fig S1B

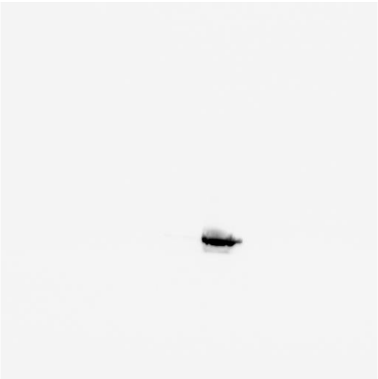

Flag

β-Actin

Fig S2A

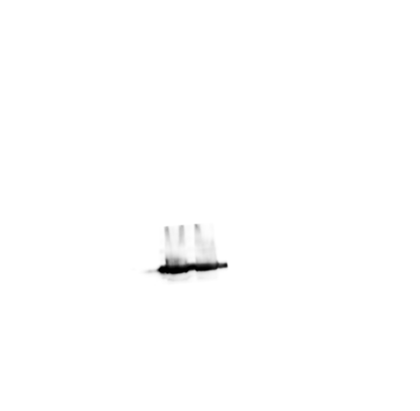

Flag

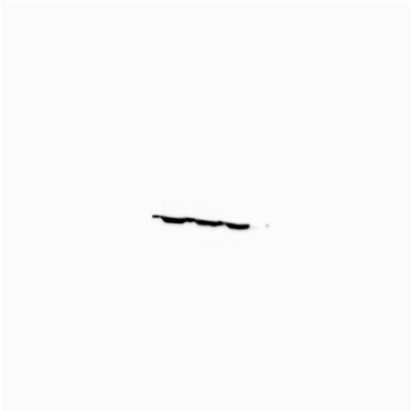

β-Actin

Fig S2B

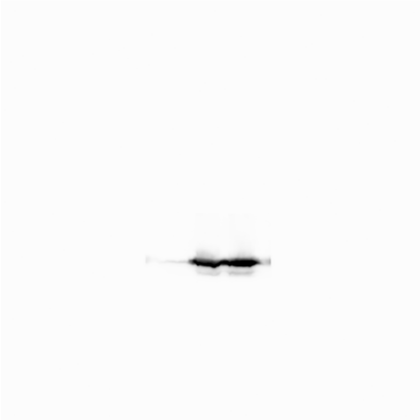

Flag

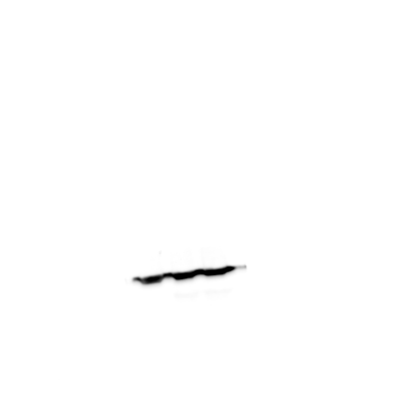

β-Actin

Fig S3A

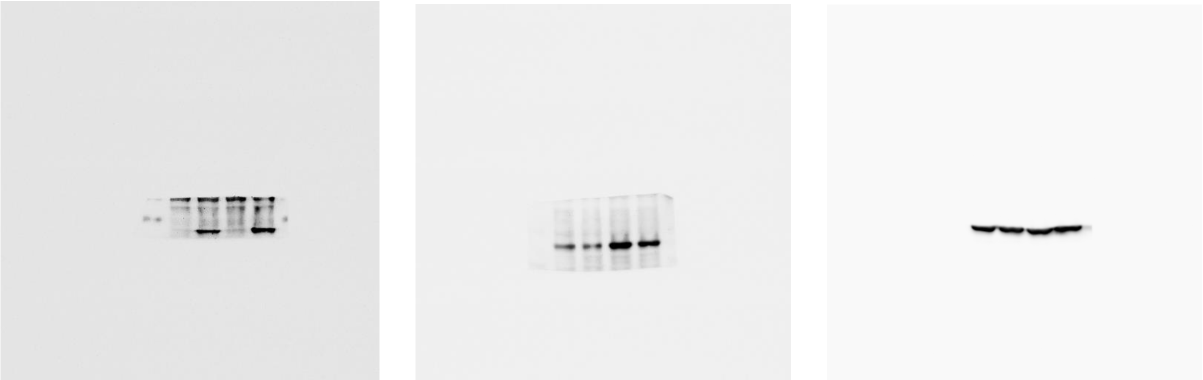

Flag P62 β-Actin

Fig S3B

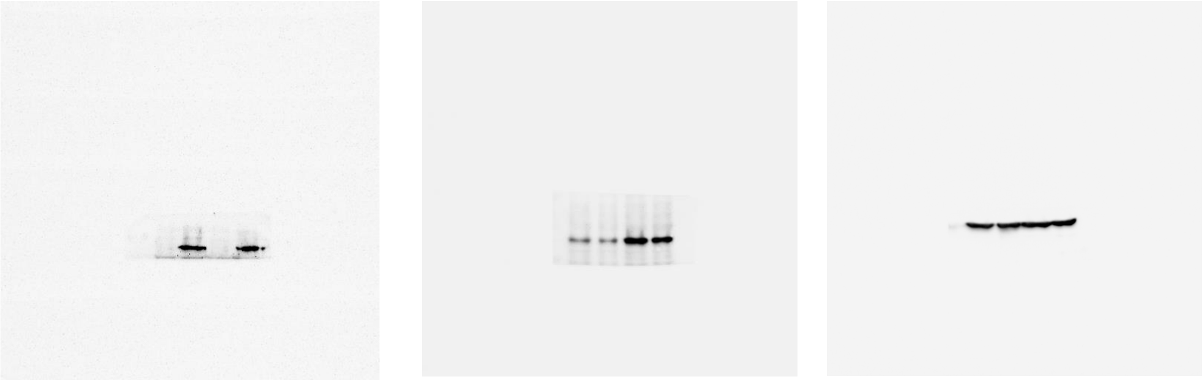

Flag P62 β-Actin

Fig S3C

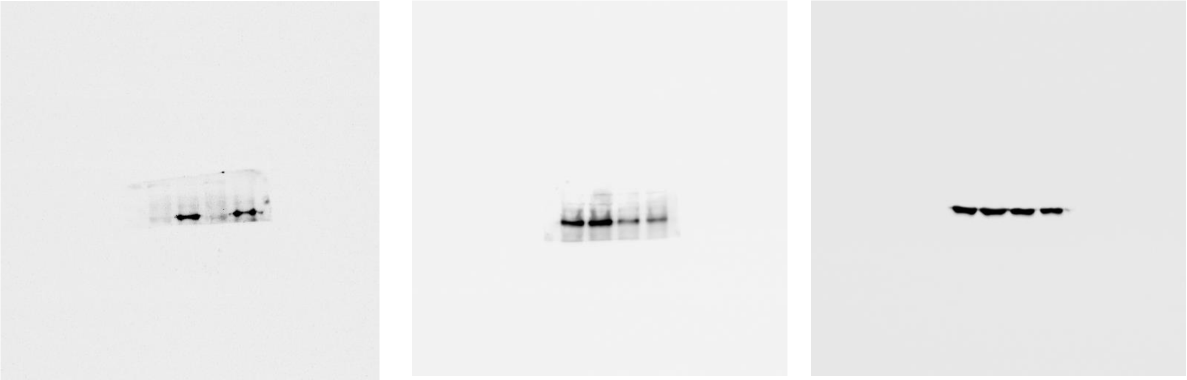

Flag BECN1 β-Actin

Fig S3D

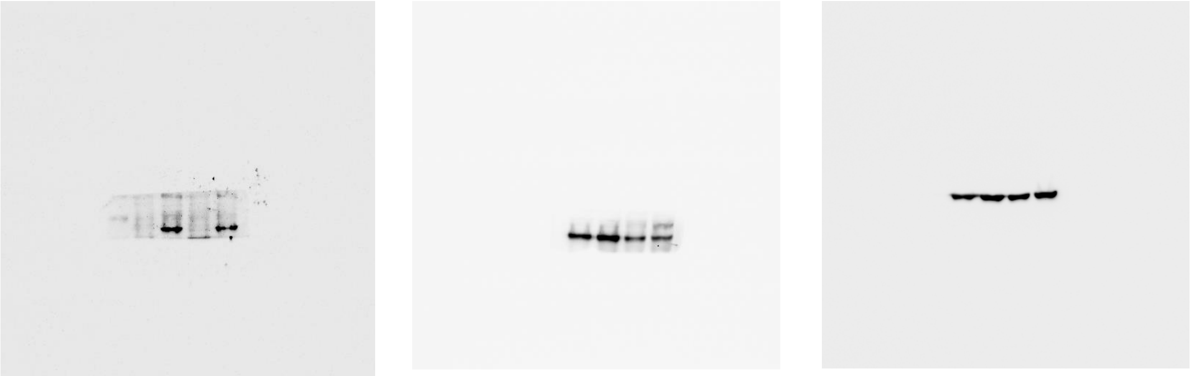

Flag BECN1 β-Actin
